# Adaptation of the Porcine Pituitary Transcriptome, Spliceosome and Editome during Early Pregnancy

**DOI:** 10.3390/ijms24065946

**Published:** 2023-03-21

**Authors:** Karol G. Makowczenko, Jan P. Jastrzebski, Marta Kiezun, Lukasz Paukszto, Kamil Dobrzyn, Nina Smolinska, Tadeusz Kaminski

**Affiliations:** 1 Department of Animal Anatomy and Physiology, Faculty of Biology and Biotechnology, University of Warmia and Mazury in Olsztyn, Oczapowskiego 1A, 10-719 Olsztyn, Poland; 2 Department of Plant Physiology, Genetics and Biotechnology, Faculty of Biology and Biotechnology, University of Warmia and Mazury in Olsztyn, Oczapowskiego 1A, 10-719 Olsztyn, Poland; 3 Department of Botany and Nature Protection, Faculty of Biology and Biotechnology, University of Warmia and Mazury in Olsztyn, Plac Lodzki 1, 10-719 Olsztyn, Poland; 4 Department of Zoology, Faculty of Biology and Biotechnology, University of Warmia and Mazury in Olsztyn, Oczapowskiego 5, 10-719 Olsztyn, Poland

**Keywords:** transcriptome, RNA-Seq, pig, pituitary gland, adenohypophysis, genes expression, lncRNA, alternative splicing, allele-specific expression, RNA editing

## Abstract

The physiological mechanisms of the porcine reproduction are relatively well-known. However, transcriptomic changes and the mechanisms accompanying transcription and translation processes in various reproductive organs, as well as their dependence on hormonal status, are still poorly understood. The aim of this study was to gain a principal understanding of alterations within the transcriptome, spliceosome and editome occurring in the pituitary of the domestic pig (*Sus scrofa domestica* L.), which controls basic physiological processes in the reproductive system. In this investigation, we performed extensive analyses of data obtained by high-throughput sequencing of RNA from the gilts’ pituitary anterior lobes during embryo implantation and the mid-luteal phase of the estrous cycle. During analyses, we obtained detailed information on expression changes of 147 genes and 43 long noncoding RNAs, observed 784 alternative splicing events and also found the occurrence of 8729 allele-specific expression sites and 122 RNA editing events. The expression profiles of the selected 16 phenomena were confirmed by PCR or qPCR techniques. As a final result of functional meta-analysis, we acquired knowledge regarding intracellular pathways that induce changes in the processes accompanying transcription and translation regulation, which may induce modifications in the secretory activity of the porcine adenohypophyseal cells.

## 1. Introduction

It is commonly known that the pituitary gland is one of the most important endocrine organs in eutherians, which participates in the maintenance of systemic homeostasis and the regulation of numerous physiological processes [1]. The pituitary gland is composed of two main structures: the anterior pituitary (adenohypophysis—AP) and the posterior pituitary (neurohypophysis—NP). The adenohypophyseal cells are capable of producing six major hormones, including adrenocorticotropin hormone (ACTH), follicle-stimulating hormone (FSH), growth hormone (GH), luteinizing hormone (LH), prolactin (PRL) and thyroid-stimulating hormone (TSH) [2]. The pituitary gland is the central part of the functional endocrine axes, including the hypothalamic–pituitary–gonadal axis (HPG), hypothalamic–pituitary–adrenal cortex axis (HPA) and hypothalamic–pituitary–thyroid axis (HPT).

The porcine HPG axis has been well-described. In the domestic pig (*Sus scrofa domestica* L.), the hypothalamic nuclei produce gonadotropin-releasing hormone (GnRH), which is transported through the hypophyseal portal system to the gonadotroph cells of AP. GnRH is bound by GnRHR, which triggers the production of the gonadotrophic hormones, LH and FSH, which are crucial for the controlling ovarian functions [3]. LH is transported to the ovaries, where it initiates ovulation and maintains corpus luteum functions. FSH activates the growth of ovarian follicles and estrogens synthesis and triggers the release of inhibin, which, together with steroid hormones, initiates feedback regulation of the HPG axis.

Adaptation of the anterior pituitary cells to peri-implantation hormonal milieu may be evidenced by the decrease in secreted LH on day 15–16 of gestation relative to day 10–12 of the estrous cycle, with no change in circulating estradiol concentrations [4]. On the other hand, other research teams reported a statistical lack of change in LH secretion between the mentioned physiological periods [5,6]. There are no reliable data on the concentrations of circulating FSH in gilts during the peri-implantation period, but many authors conclude its basal concentration [7,8,9], which would also be associated with a lack of change in the secretion of this hormone relative to the mid-luteal phase of the estrous cycle [10,11]. Despite the lack of data on changes in circulating ACTH concentrations in gilts at the early stage of gestation compared to the estrous cycle, the worth noting is a reduction in circulating ACTH levels in the corresponding stages observed in women and rats [12,13]. In addition, we would like to point out that despite many factors produced by other glands, many hormones produced locally in the pituitaries also act in an auto- or paracrine manner, participating in the AP control [14,15,16,17,18].

According to Ing [19], hormonal status directly affects the gene expression profile in a tissue-specific manner and in the specific physiological periods. For example, the expression of genes coding adipokines (i.e., adiponectin, chemerin and leptin) and their receptors in the porcine pituitary are influenced by the hormonal milieu that is specific for different pregnancy periods and phases of the estrous cycle [20,21,22]. Recently, Xiong et al. [23] showed that noncoding RNAs can significantly affect intracellular signaling in the porcine AP cells. However, the cited studies contribute only partial information on the subject and do not involve a broader approach to the changes that take place within the AP during significant events such as the implantation process. Extensive transcriptomic studies have already been carried out, explaining physiological processes in the porcine reproductive organs [24,25,26,27], and two research studies have described transcriptomic changes in the gilt pituitary gland anterior lobe (using the complementary DNA—cDNA microarray technique) [28,29], which controls the reproductive system.

During the embryo implantation stage of pregnancy, about 30% of embryos are lost [30]. Therefore, this is a crucial period for the reproductive success of pigs. To date, the impact of the embryo implantation on the gene expression profile and other associated transcriptomic processes occurring within the pituitary cells has not been analyzed in detail in any organism, and the available literature is insufficient to conclusively deduce the impact of variable hormonal milieu on the transcriptomic mechanisms occurring within cells. During the embryo implantation period (EP; days 15–16 of pregnancy), the fully developed and functional corpus luteum has similar secretory activity, as in the mid-luteal phase (ML; days 10–12 of the estrous cycle) [5,6]. It seems that analysis of only secretory profiles of pituitary gonadotropins and ovarian steroid hormones do not explain all changes taking place in the pituitary cells during early pregnancy. Therefore, in-depth studies are needed, also at the transcriptomic level, to detect modifications of physiological processes occurring in the AP cells during this critical period of pregnancy. We hypothesized that there are differences between the transcriptomic (including differentially expressed genes—DEGs; differentially expressed long noncoding RNAs—DELs; allele-specific expression—ASEs), spliceosomal (differential alternative splicing events—DASs) and editomal (RNA editing process alterations) profiles of the pituitary cells isolated from gilts in the mentioned physiological states. To test this hypothesis, we applied molecular biology technology—high-throughput transcriptome sequencing (RNA-Seq)—and comprehensive bioinformatic analyses of the obtained results. The aim of this experiment was to provide detailed knowledge on the changes in transcriptome, spliceosome and editome, taking place within the adenohypophyseal cells isolated during the implantation period of pregnancy and the mid-luteal phase of the estrous cycle, when numerous factors that affect the activity of the pituitary gland are radically altered.

## 2. Results

### 2.1. Overall Statistics of RNA-Seq Data Mapping

The cumulative number of raw reads obtained by high-throughput transcriptome sequencing of all samples was 714,796,216, and the total number of processed reads was 560,997,084. The overall number of processed reads mapped to the reference *S. scrofa* genome ver. 11.1.104 was 544,422,058. Of them, 91.34% were mapped uniquely. Detailed numerical data are summarized in Table 1. Files containing the raw reads obtained as the sequencing result were deposited in the European Nucleotide Archive (ENA) under accession number PRJEB56673, and the processed reads mapping output files were provided to the ArrayExpress database under accession number E-MTAB-12316 (detailed in the Data Availability Section).

### 2.2. Differentially Expressed Genes

**Figure 1:**
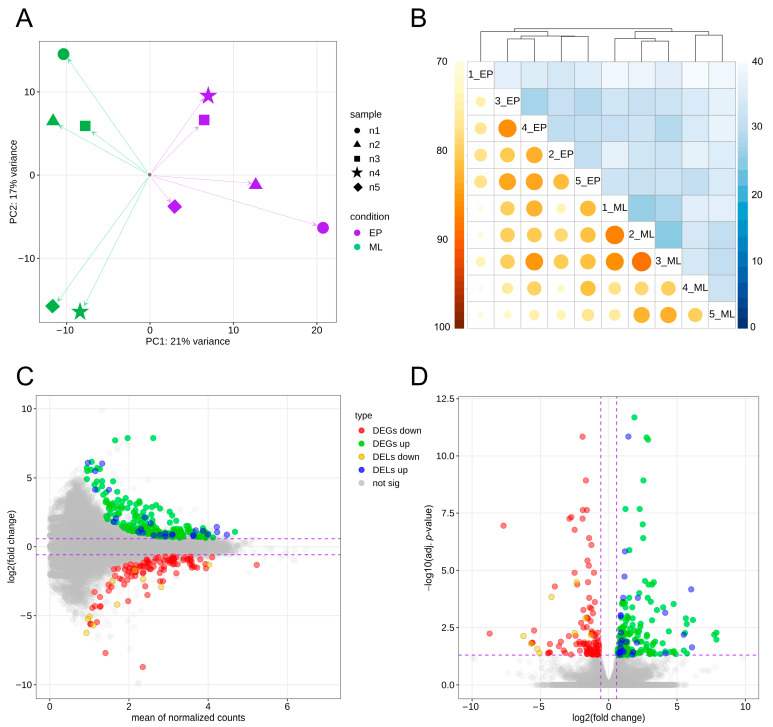
(**A**) Principal component analysis (PCA) of all differentially expressed genes. (**B**) Cluster analysis based separately on the Euclidean distance measure (blue part of the matrix) and Pearson correlation (orange part). The dendrogram characterizes the sample arrangement obtained in both analyses. (**C**) MA plot showing the mean of normalized counts values against log_2_(fold change) for DEGs and DELs comparing EP and ML groups. (**D**) Volcano plot presenting the log_2_(fold change) plotted against −log_10_(adjusted *p*-value) for DEGs and DELs between EP and ML libraries. The purple dashed lines indicate cut-off thresholds detailed in the text. Abbreviations: DEGs—differentially expressed genes, DELs—differentially expressed long noncoding RNAs, EP—the embryo implantation period of pregnancy, ML—the mid-luteal phase of the estrous cycle, not sig—not significant.

**Figure 2:**
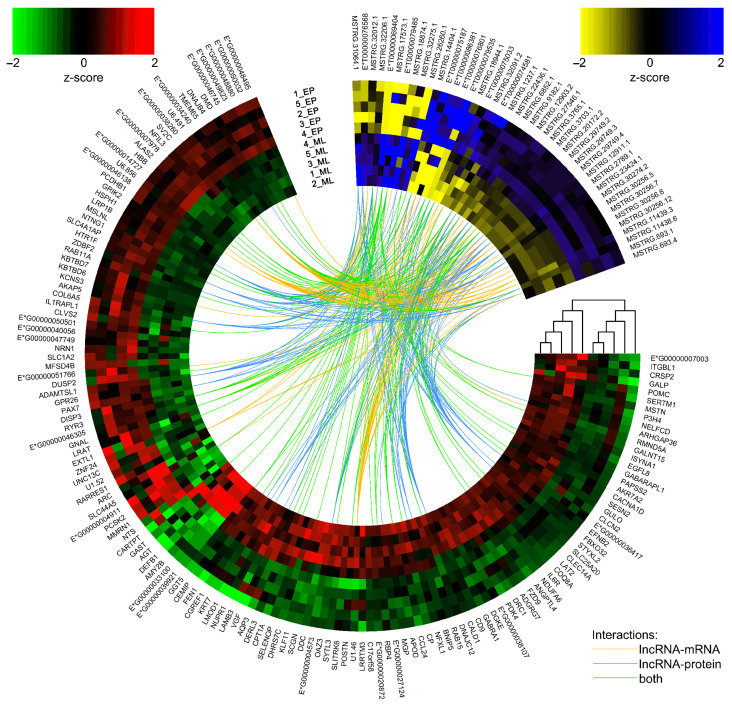
Visualization of *trans*-interactions occurring between differentially expressed protein-coding genes (DEGs) and differentially expressed long noncoding RNAs (DELs) within the porcine adenohypophyseal cells isolated during the embryo implantation pregnancy period (EP) in comparison to the mid-luteal phase of the estrous cycle (ML). Heatmaps show the expression profiles of DEGs (red–green) and DELs (blue–yellow). The sample layout in the heatmaps was created based on the DEGs cluster analysis, as shown using a dendrogram. Both DEGs and DELs were arranged according to increasing log_2_(fold change). Details of the interactions are provided in the text. The “NSSSC” fragments of Ensembl numbers were abbreviated with “*”.

Principal component analysis (PCA) and cluster analyses, based on the sample-to-sample computations of Euclidean distances and Pearson correlations, revealed a significant level of gene expression profiles variation between EP and ML groups and high homogeneity within these groups (Figure 1A,B). Expression profile analysis conducted with the DESeq2 tool showed significant expression differences (adjusted *p*-value < 0.05) of 332 transcriptionally active regions (TARs) within EP group compared to ML samples, among which 147 DEGs were detected. Among all identified DEGs, 63 were upregulated within EP samples, while 84 were downregulated (Table S1). Calculated binary logarithm of fold change (FC) values of DEGs ranged from 5.71 (*NRN1*; short gene names are explained in the Abbreviations Section) to −5.62 (*BNIP5*). The expression profiles of all DEGs and DELs (described later in the text) were presented by normalized counts, log_2_(FC) and −log_10_(adjusted *p*) values in MA and volcano plots (Figure 1C,D). A heatmap of the DEGs’ expression profiles is shown in Figure 2 later in this paper.

### 2.3. Long Noncoding RNA Identification and Cis-/Trans-Acting on Protein-Coding Genes

Identification of novel long noncoding RNAs (lncRNAs) was performed by a series of preprocessed RNA-Seq data filtering steps. Initially, transcript sequences marked in the Ensembl database [31] as “protein-coding biotype” were removed from the dataset, as well as RNA molecules that were shorter than 200 nt and mono-exonic. The result of these steps was 16,772 potential lncRNA candidates, including 1660 molecules marked in the Ensembl database as ‘lncRNA biotype’. The unlabeled sequences were analyzed for coding potential using five programs: the coding-noncoding index (CNCI) [32], the coding potential assessment tool (CPAT) [33], the coding potential calculator 2 (CPC2) [34], the flexible extraction of lncRNAs (FEELnc) [35] and the predictor of lncRNAs and mRNAs based on an improved *k*-mer scheme (PLEK) [36], which allowed us to narrow down the novel lncRNAs pool to 2443 molecules. Of these, 2289 sequences survived the alignments to the protein family database (Pfam) [37] performed with the HMMER tool [38] and to the small RNA model records of the RNA families (Rfam) database [39] with the Infernal cmscan software [40]. The results of the seven mentioned programs are summarized as the Venn diagrams in Figure S1.

The differential expression analysis performed on the combined dataset of newly discovered lncRNAs and macromolecules previously reported in the Ensembl database revealed the alteration of 43 molecules’ expression (encoded by 37 genes) in the EP group relative to ML samples. Within the DELs set, 9 lncRNAs were previously known, and the remaining 34 were identified in this study. The expression of 33 DELs was upregulated and 10 were downregulated in the EP probes compared to ML group (Table S2). Calculated DELs’ log_2_(FC) values ranged from 6.09 (*ENSSSCT00000079535*) to −6.24 (*ENSSSCT00000069404*). A visualization of the statistical significance cut-offs and expression differences applied during DELs analysis is summarized in Figure 1C,D.

*Trans*-interaction analysis revealed 2313 co-expression events between all DELs and 146 DEGs (assuming a strong Pearson correlation). Following messenger RNA (mRNA)–lncRNA binding (LncTar tool [41]) and protein–lncRNA spatial matching (lncPro tool [42]), analyses revealed 189 and 1632 relationships, respectively. The macromolecule matching results allowed us to limit the number of detected *trans*-interactions to 295 events associated with 42 DELs and 71 DEGs (Table S3). In the narrowed dataset, 165 interactions showed a positive correlation, whereas 130 events showed a negative correlation. All of the described interactions based on both co-expression of DEL–DEG pairs, including the heatmaps presenting DEGs and DELs expression profiles within all samples, are visualized in Figure 2. *Cis*-interaction analysis identified 74 DEL–gene pairs. However, it did not identify any significant DEL–DEG pair. Therefore, data on the protein-coding genes and DELs co-localization were not included in further functional analyses.

### 2.4. Differential Alternative Splicing Events of Differentially Expressed Genes

**Figure 3:**
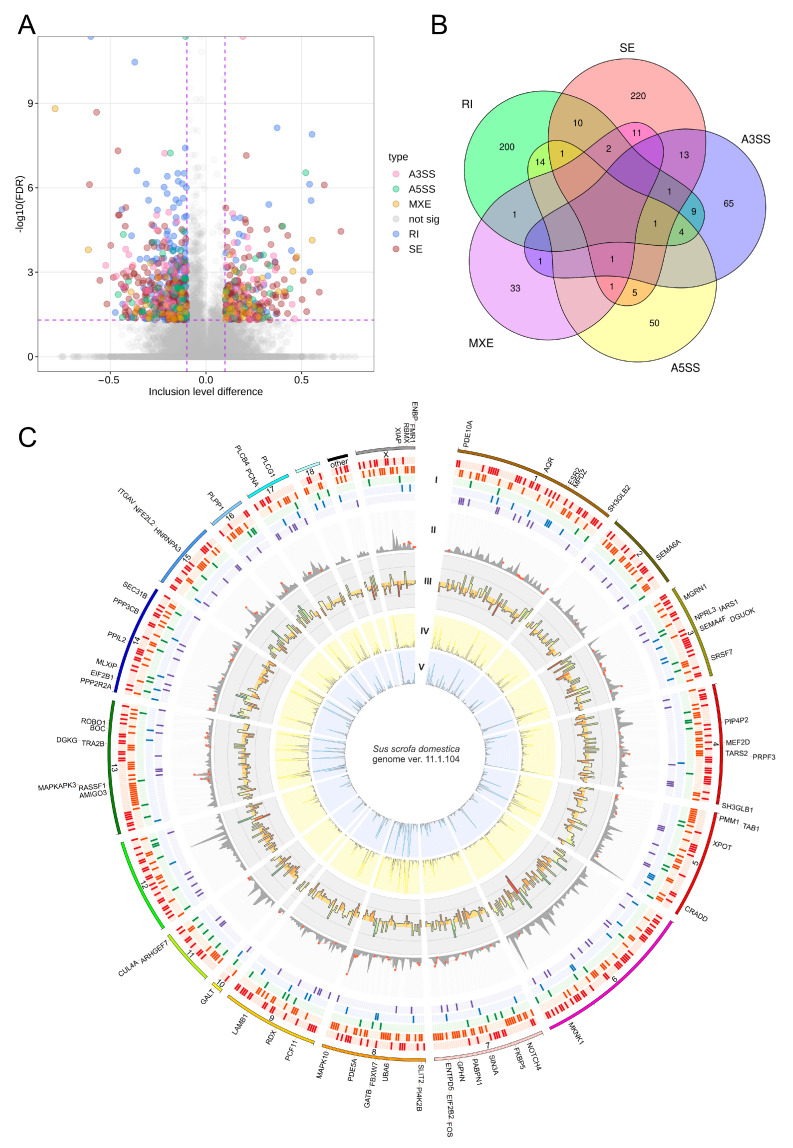
(**A**) Volcano plot showing inclusion levels differences against the statistical significance of DASs identified within genes of porcine adenohypophyseal cells during EP compared to ML. The purple dashed lines indicate the applied cut-off thresholds, described in the text. (**B**) The Venn diagram characterizing the occurrence of distinct types of identified DASs within genes. (**C**) Circular chart visualizing the distribution within the domestic pig genome of identified DASs within porcine adenohypophyseal cells between EP and ML. The subsequent circles characterize the parameters of the identified DAS events: I—the occurrence within genes of identified DASs classified into five types: SE marked with red stripes, RI marked with orange stripes, A5SS marked with green stripes, MXE marked with blue stripes and A3SS marked with purple stripes; II—statistical significance expressed as −log_10_(false discovery rate) and positions of DAS-containing genes involved in at least one of the 50 KEGG pathways obtained during the functional analyses; III—inclusion level differences of identified DASs; IV—the summarized level of inclusions in EP samples; V—the summarized level of inclusions in ML samples. Abbreviations: A3SS—alternative 3′ splice site, A5SS—alternative 5′ splice site, DASs—differential alternative splicing events, EP—the embryo implantation period of pregnancy, ML—the mid-luteal phase of the estrous cycle, MXE—mutually exclusive exons, not sig—not significant, RI—retained intron, SE—skipping exon.

**Figure 4:**
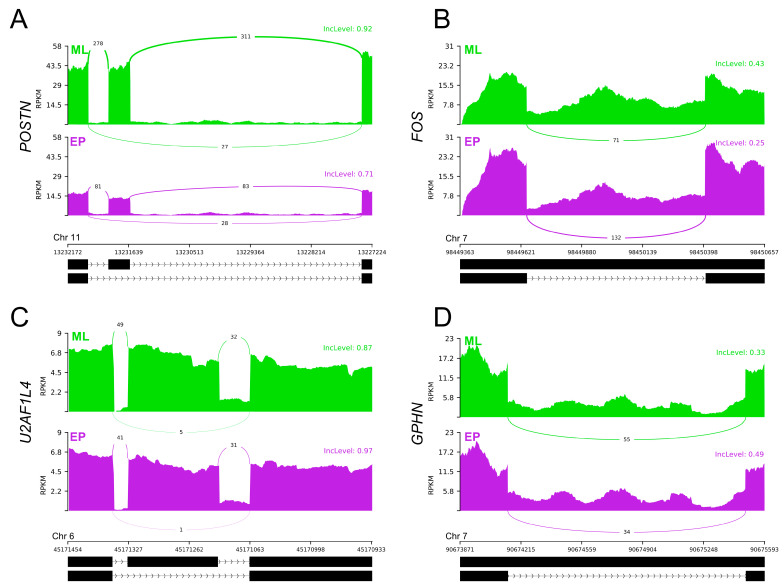
Sashimi plot visualizing the detected coverage of RNA-Seq reads on the reference genome and the average values of reads combining distant genome fragments (black blocks underneath the graphs) in EP and ML groups. Displayed fragments of (**A**) *POSTN*, (**B**) *FOS*, (**C**) *U2AF1L4* and (**D**) *GPHN* genes were classified as statistically significant differentiated alternative splicing events. Abbreviations: Chr—chromosome, EP—the embryo implantation period of pregnancy, IncLevel—inclusion level, ML—the mid-luteal phase of the estrous cycle, RPKM—reads per kilobase million.

The adopted procedure, incorporating the replicate multivariate analysis of transcript splicing (rMATS) software [43], allowed for the detection of 52,872 alternative splicing events, including 784 DASs resulting from the comparison of EP vs. ML samples (Figure 3). Among all detected DASs, 78 were classified as alternative 5′ splice site (A5SS), 100 as alternative 3′ splice site (A3SS), 55 as mutually exclusive exons (MXE), 272 as retained intron (RI) and 279 as skipping exon (SE). Calculated inclusion level difference values ranged from 0.79 (MXE) to −0.71 (SE), both within *CELF4*. All disclosed DASs are localized within 643 genes, including 19 lncRNA-coding genes. Alternative splicing events were discovered in the 7 DEGs regions: *CACNA1D* (RI), *DERL3* (SE), *DNAJB4* (SE), *EXTL1* (RI), *GALP* (RI), *GRIK2* (RI) and *POSTN* (SE). Selected events of alternative splicing occurring within the *FOS*, *GPHN*, *POSTN* and *U2AF1L4* genes are visualized in Figure 4, while all identified cases are summarized in Table S4.

### 2.5. Single Nucleotide Variant Calling, Allele-Specific Expression Variations and RNA Editing

During analyses of 10 RNA-Seq libraries’ data, a total of 701,422 single nucleotide variants (SNVs) were called for the porcine AP transcriptome using the rMATS discovery of differential variants in RNA (rMATS-DVR) software [44]. After applying the standard genome analysis toolkit (GATK) [45] filtering parameters, 651,675 polymorphic sites were retained during the first SNVs quality processing step. Subsequently, the filtering step involving the location of variants in the genome elements (within bidirectional genes, simple sequence repeats or close proximity of splice junction sites or paralog regions) depleted the dataset to 237,314 SNVs. During the following filtration step, 3082 SNVs closely located and labeled as pairs and 789 as triplets were detected and filtered out. Finally, the processed dataset 97,094 SNVs having a non-zero expression value in more than half of the samples remained.

Among the processed variants, 20,208 sites showed significant differences in alternative allele fraction (∆AAF) between the EP and ML groups and reached the assumed statistical significance threshold, resulting in a preliminary classification as ASEs. The statistical significance assumptions of the ꭕ^2^ control test were fulfilled by 8729 ASEs. Of these, the occurrence of 8175 ASEs was found within protein-coding transcripts, 297 in lncRNA transcripts, 47 within other noncoding RNAs (such as small nucleolar RNAs, small nuclear RNAs and micro RNAs) and 1 each within ribozyme and mitochondrial tRNAs. The remaining ASEs occurred in pseudogene regions and uncharacterized parts of the porcine genome. Due to the ASEs occurrence across protein-coding genes elements, they were subdivided into localization classes: upstream variants (207 ASEs), 5′ UTR variants (154), CDS variants (synonymous—1277 and missense—377), intron variants (4183), 3′ UTR variants (1283) and downstream variants (694). According to the results of the sorting intolerant from the tolerant (SIFT) [46] module of the variant effect predictor (VEP) tool [47], the biological effect of 341 ASEs is tolerated (or tolerated with low confidence) and the effect 36 is deleterious (or deleterious with low confidence). The detrimental effect of ASEs has been identified in the 29 genes: *AKAP11*, *ALDH3A2*, *ATRX*, *BLTP2*, *BMS1*, *BTAF1*, *CAVIN2*, *CCP110*, *CD47*, *CNST*, *DISP1*, *DMXL1*, *ELP1*, *GMPS*, *IGSF1*, *LSR*, *MAGI1*, *MAP1A*, *MIGA1*, *NDUFA10*, *PARG*, *PTPRN2*, *RTN4*, *SIL1*, *SUCLA2*, *TAOK2*, *TENM3*, *TMED3* and *URGCP*. Additionally, 4619 ASEs were classified as ‘HeteroAlt’, 3375 as ‘HeteroRef’ and 735 as ‘true’ heterozygotes, due to its reference/alternative imbalance ratio.

**Figure 5:**
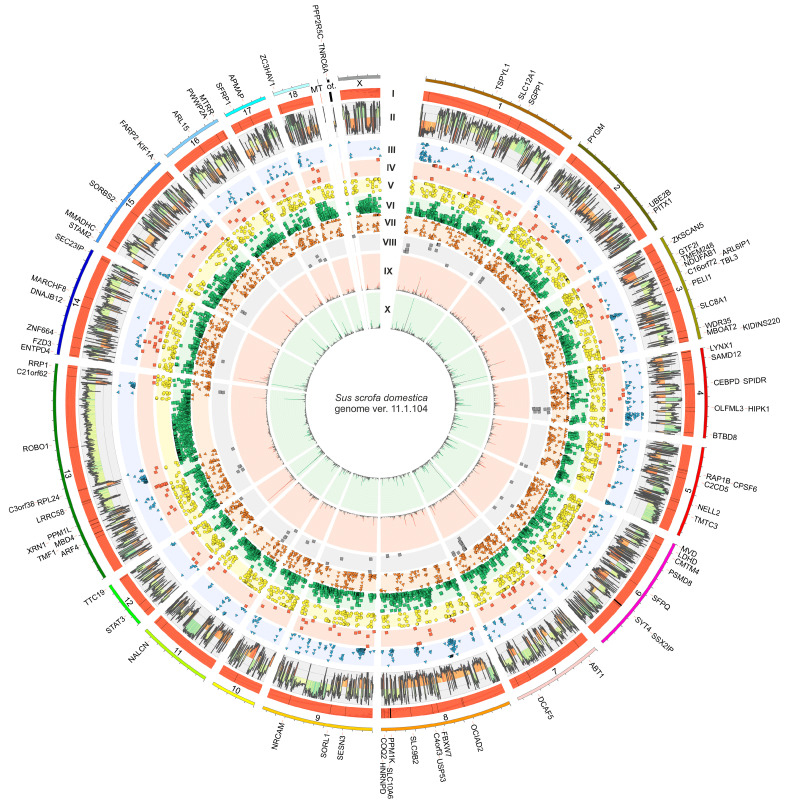
Visualization of the distribution within the domestic pig genome of identified allele-specific expression variants (ASEs) and RNA editing events alterations differentiating within porcine adenohypophyseal cells during the embryo implantation period (EP) compared to the mid-luteal phase of the estrous cycle (ML). The subsequent circles present the following data: I—distribution of ASEs (red bars) and RNA editing events (black stripes) on the porcine chromosomes, II—histogram of differences in alternative allele fraction (∆AAF) values for identified events, III—sites localized within the intergenic regions, IV—sites localized within the 5′ untranslated regions (UTRs), V—sites localized within the coding DNA sequences (CDS), VI—sites localized within the intronic regions, VII—sites localized within the 3′ UTRs, VIII—sites localized within the noncoding regions, IX—histogram of sum of alternative allele coverage within EP samples and X—histogram of the sum of alternative allele coverage within ML samples. For circles III–VIII, the distance from the circle’s center symbolizes the significance level, expressed as −log_10_(false discovery rate). Gene names on the outside of the circle mark the positions of identified RNA editing events.

The 5072 polymorphic sites were assigned to RNA editing canonical substitutions (A-to-I or C-to-U). Of these, 1425 sites passed the incomplete abundance of alternative variant criterion (AAF < 0.7 across all samples). The 122 polymorphic sites were qualified as RNA editing candidates, due to the close proximity of PRE-1 SINE regions in the porcine genome. According to their localization in the porcine genome elements, identified RNA editing events were classified as follows: upstream variants (6 sites), 5′ UTR variants (4), CDS variants (synonymous—21 and missense—1), intron variants (38), 3′ UTR variants (30), downstream variants (17), intergenic variant (1) and noncoding transcript exon variants (4). In total, 52 of the detected RNA editing events intersected with the database of pig RNA editing sites (PRESDB) [48] records, including 12 brain-specific sites and 4 previously identified in the swine reproductive organs (ovary and uterus). These 16 RNA editing events with known occurrence organs were found in 15 genes: *C16orf72*, *C2CD5*, *FZD3*, *GTF2I*, *LDHD*, *MARCH8*, *MMADHC*, *OCIAD2*, *SEPTIN2*, *SLC9B2*, *STAM2*, *STAT3*, *TMF1*, *ZKSCAN5* and *ZNF664*. The ∆AAF value for the detected RNA editing events ranged from 0.66 to −0.64. Data on the prevalence of ASEs and RNA editing process alterations in the pig genome are summarized in Figure 5, and Tables S5 and S6, respectively.

### 2.6. Functional Annotation of Target Protein-Coding Genes

**Figure 6:**
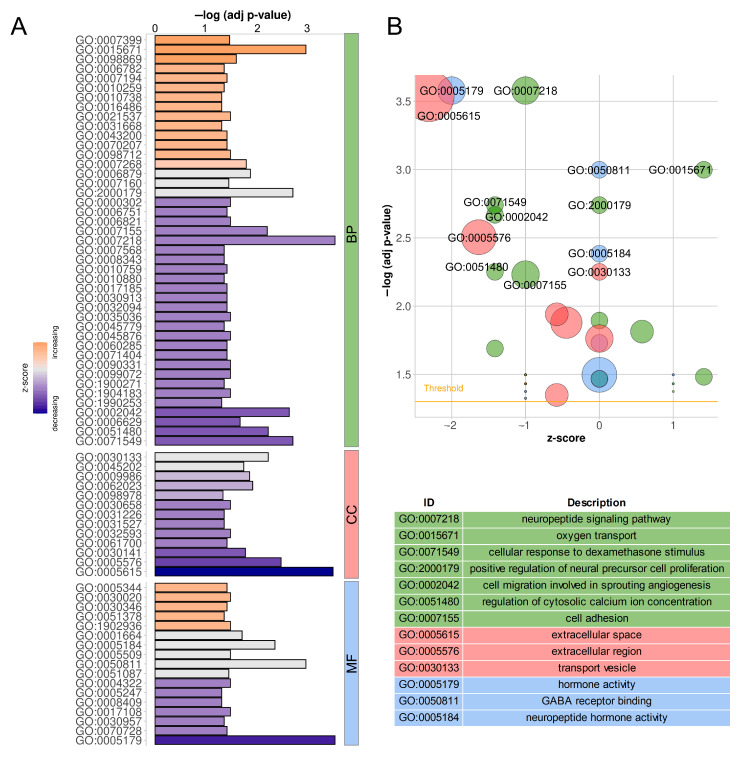
Summary of GO terms enrichment analysis with differentially expressed genes (DEGs). (**A**) The bar plot represents the ratio between up- and downregulated DEGs. Orange bars represent GO terms with the advantage of upregulated DEGs, whereas purple bars show terms that consist of downregulated DEGs, mainly. The GO terms are divided into biological process (BP), cellular component (CC) and molecular function (MF) aspects. The length of the bar indicates the individual terms’ statistical significance levels, obtained during the functional analysis. (**B**) The bubble chart shows the dependence of the normalized ratio of up- and downregulated DEGs (z-score) on the statistical significance level of GO terms. The size of the bubble is proportional to the DEGs abundance identified in the study, classified by the term. The names of the selected GO terms are listed in the table below.

In order to summarize and combine the results obtained for all five transcription-related phenomena, an extended functional meta-analysis was performed. The Ko-based annotation system (KOBAS) ver. 3.0 [49] was applied to enrich the Gene Ontology Consortium (GO) [50,51] and the Reactome Knowledgebase [52] categories, as well as pathways in the Kyoto Encyclopaedia of Genes and Genomes (KEGG) [53] separately for each phenomenon, based on directly related protein-coding genes. The GO enrichment analysis of DEGs; DEGs *trans*-interacting with DELs; and genes containing DASs, ASEs and/or modifications of RNA editing showed a significant contribution of 50, 23, 230, 1016 and 32 genes encompassing 43, 35, 48, 66 and 73 biological process (BP) terms; 17, 17, 25, 25 and 17 molecular function (MF) terms; and 13, 6, 23, 29 and 11 cellular component (CC) terms (Table S7), respectively,. Tendencies in the DEGs’ impact on the genes’ ontology are visualized in Figure 6. The Reactome-based analysis demonstrated the significant enrichment of 12 terms by DEGs, 12 terms by DEGs *trans*-interacting with DELs, 29 terms by genes containing DASs, 3 terms by genes containing ASEs and 8 terms by genes containing RNA editing modifications (Table S7).

Detected DEGs, DELs, DASs, ASEs and modifications of RNA editing were grouped into clusters corresponding to KEGG pathway membership, and statistical analysis of the regrouped data was again performed. The assumed recalculated statistical significance threshold was met by 79 metabolic or signal transduction pathways. The resulting pool included pathways related to pathological processes, such as autoimmune diseases, carcinogenesis and infections caused by various pathogens, which were not analyzed due to their lack of relevance to the investigated tissue and this research’s main objective. After refocusing on physiological processes, the resulting collection consisted of 50 KEGG pathways (Table 2). Maps summarizing the contribution of the phenomena analyzed are presented in Figures S2–S19. Additionally, important for AP functioning, the ‘Calcium signaling pathway’ (ssc04020, Figure S20) and the ‘JAK/STAT signaling pathway’ (ssc04630, Figure S21) that did not reach the assumed statistical significance threshold (for both false discovery rate—FDR = 0.004) of the KEGG enrichment process were examined.

### 2.7. Quantitative Real-Time PCR and PCR Validations

**Figure 7:**
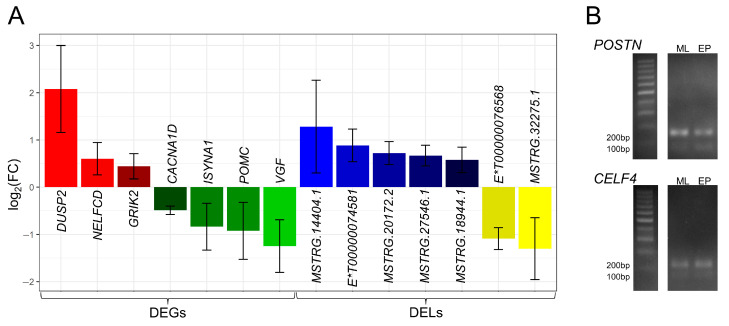
(**A**) Quantitative real-time PCR validations of RNA-Seq results performed for DEGs—*CACNA1D*, *DUSP2*, *GRIK2*, *ISYNA1*, *NELFCD*, *POMC* and *VGF*—and DELs—*ENSSSCT00000074581*, *ENSSSCT00000076568*, *MSTRG.14404.1*, *MSTRG.18944.1*, *MSTRG.20172.2*, *MSTRG.27546.1* and *MSTRG.32275.1* with reference genes, *GAPDH* and *PPIA*. All data are expressed as log_2_(FC) ± confidence interval (n = 5). Error bars not crossing the *X*-axis indicate that the corresponding means comparison is statistically significant to 5% in the Student’s *t*-test. (**B**) The PCR validation of differential alternative splicing events classified as skipping exon within *CELF4* and *POSTN*. The PCR image shows inclusion and skipping exon levels in ML and EP groups. Abbreviations: bp—base pairs, DEGs—differentially expressed genes, DELs—differentially expressed long noncoding RNAs, EP—the embryo implantation period of pregnancy, log_2_(FC)—binary logarithm of fold change, ML—the mid-luteal phase of the estrous cycle.

To verify the obtained RNA-Seq results, two DASs classified as SE were selected for the polymerase chain reaction (PCR), and seven DEGs and seven DELs were selected for quantitative real-time PCR (qPCR). In the porcine adenohypophyseal cells acquired during the EP period compared to ML, the relative mRNA abundance of, *DUSP2*, *ENSSSCT00000074581*, *GRIK2*, *MSTRG.14404.1*, *MSTRG.18944.1*, *MSTRG.20172.2*, *MSTRG.27546.1* and *NELFCD* was upregulated, whereas mRNA level of *CACNA1D*, *ENSSSCT00000076568*, *ISYNA1*, *MSTRG.32275.1*, *POMC*, and *VGF* was downregulated (Figure 7A). All qPCR expression patterns agreed with the RNA-Seq results. The effect of comparing PCR results for the EP vs. ML groups was an apparent increase in the fraction of both *CELF4* and *POSTN* transcripts lacking the targeted exon (Figure 7B). For *POSTN*, differences in the gene expression were also evident. The PCR results agreed with the tendency calculations of exons inclusion/exclusion differences based on RNA-Seq results. Validation results confirmed the veracity and accuracy of the high-throughput methods applied in this research.

## 3. Discussion

This work is the first attempt to understand the physiological changes occurring in the porcine AP during the early stages of pregnancy, controlled by such transcriptomic phenomena as the genes and lncRNAs expression modifications, as well as changes in the alternative splicing, allele-specific expression and RNA editing. High-throughput transcriptome sequencing techniques were used to conduct the study, which, combined with advanced in silico analyses of the acquired data, provided detailed insight into the molecular mechanisms initiated primarily by changes in the peri-implantation hormonal milieu. Briefly, we identified 147 DEGs, 43 DELs that exhibited 295 *trans*-interactions with 71 DEGs, 784 DASs within 643 genes, 8729 ASEs and 122 RNA editing sites in the EP group vs. ML samples. The performed qPCR and PCR validations confirmed the bioinformatic predictions for 7 DEGs, 7 DELs and 2 SE-type DASs. Based on a functional meta-analysis, the effects of the observed transcriptomic phenomena were determined within genes whose expression products are involved in signal transduction pathways and metabolic processes relevant to the AP function of gilts during early pregnancy.

### 3.1. Reception of the HPG Axis Signals—GnRH and Estrogens

Due to the analyses of AP related to the physiological processes of the gilt reproduction, the most expected changes were in transcriptomic phenomena related to the reception by adenohypophyseal cells of signals from the higher and lower branches of the HPG axis and the preparation of the corresponding response. As expected, the functional analysis yielded a ‘GnRH signaling pathway’ (Figure S15) related to signals generated by the upstream regulatory level, and an ‘estrogen signaling pathway’ (Figure S19) depicting a feedback response of AP cells to signals from the downstream axis.

Analyzing the RNA-Seq data, we did not identify significant differences in transcriptomic phenomena occurring within the *FSHB* gene, which encodes the FSH subunit β. This may indicate a similar production of this hormone by gonadotrophs during EP and ML. Our attention was attracted by the downregulation of *VGF* gene expression during EP and *trans*-interactions at the mRNA–lncRNA level with *MSTRG.17573.1* (positive correlation of expression profiles) and protein–lncRNA level with *MSTRG.27546.1* (negative correlation). Moreover, the identified changes in the expression of *VGF* and the second DEL were confirmed by qPCR. *VGF* is implicated in fertility [54], and according to Choi et al. [55], it is involved in the homeostatic control of *FSHB* expression within gonadotrophs in an autocrine manner. This mechanism relies on strongly induced *VGF* expression by high-frequency GnRH stimulation, in effect producing and secreting the NERP1 protein, which in turn suppresses *FSHB* expression in a concentration-dependent pattern. Thus, the preferential induction of *FSHB* expression in the gonadotrophs only occurs during the reception of low-frequency GnRH pulses. What is extremely interesting, according to Fletcher et al. [56], is that *VGF* expression is specific to female AP-building gonadotrophs. The aforementioned modifications of *VGF* expression are most likely part of this self-control mechanism, leading to a constant serum FSH level in gilts during the embryo peri-implantation period. The functioning of this homeostatic mechanism is substantiated by the maintenance of a constant concentration of circulating FSH on days 12–25 of gestation in gilts [57]. Moreover, in the EP group, we identified a downgrade of the A3SS-class DAS event, involving a decrease in the fraction of *GALT* transcripts, into which a STOP signal is inserted into the CDS just before the third exon. In the APs, the GALT enzyme can modulate the production of FSH various glycoforms, affecting its biological activity. Increased expression of this gene has been reported in the rat APs during proestrus and estrus [58]. Enhanced production of active GALT macromolecules may be an additional mechanism regulating the bioactivity of secreted FSH in gilts at early stages of gestation.

The *ESR2* gene (also referred to as *ERβ*) encodes a classic estrogen receptor protein 2 whose molecules are located in the nucleus and cytoplasm [59]. After ligand attachment with a C-terminal domain and homo- or heterodimerization, it can bind to a specific DNA sequence with an N-terminal domain initiating the transcription process. *ESR2* expression has been confirmed in the porcine APs [60]. ESR2 macromolecules, present in the pituitary, are suspected to be involved in a negative feedback loops in the studied periods, during which estradiol inhibits GnRH-induced LH secretion [61]. There is a discrepancy between *ESR2* mRNA expression levels and measured protein levels [62], which in human, Japanese macaque and rats is explained by the occurrence of an alternative splicing mechanism for the first untranslated exon, caused by the presence of multiple independent promoter sequences for this gene [63,64]. Moreover, estradiol would be involved in controlling this process, i.e., in shaping the 5′ UTR sequence of the transcripts [64]. In the analyzed transcriptomes of the porcine AP cells, we identified an event classified as alternative splicing manifesting as a decrease in the *ESR2* transcripts containing the first exon fraction in the EP group relative to ML. Smith et al. [64] pointed out that the transcript variant containing the shortest 5′ UTR of 90 nt manifested the least inhibitory effect on ESR2 translation. The transcript variant promoted in our experiment has a 5′ UTR of 93 nt that allows us to assume the higher concentrations of ESR2 protein in the porcine AP cells during early pregnancy. This transaction-controlled mechanism may be involved in the regulation of LH secretion by the gilt AP cells during the EP phase, perhaps taking part in the mentioned changes reported by Guthrie et al. [4].

### 3.2. Reception of Hypothalamic Neurotransmitters

An exceptionally interesting observation seems to be the predicted modification of the activity of neurotransmitter-initiated signal transduction pathways. Functional meta-analyses have revealed four main pathways significantly affected by the transcriptomic phenomena analyzed in this research: cholinergic (Figure S8), dopaminergic (Figure S13), GABAergic (Figure S14) and glutamatergic (Figure S11). The presence of each of the four neurotransmitters (acetylcholine, dopamine, γ-aminobutyric acid—GABA and glutamate) was described in hypothalamic-portal blood [65,66,67,68], and the presence of their various receptors (both metabotropic G protein-coupled and ion-gated channels) was detected in adenohypophyseal endocrine cell sub-populations [69]. Moreover, it has been proven that acetylcholine, GABA and glutamate can be produced by the AP cells and locally act in an auto- and paracrine manner [69].

In our opinion, the most important changes are contained within the GABA signal reception and internalization. In the big picture, GABA, acting on the AP hormone-secreting cells, induces the increase of ACTH, GH and LH (and most likely FSH); induces TSH secretion; and inhibits basal PRL release [70,71,72]. In addition, the activation of GABA receptors may lead to Cl^−^ efflux, activation of voltage-gated Ca^2+^ influx [71]. In the research set-up analyzed, we identified the lower expression of Cl^−^-conducting receptor α1 subunit gene—*GABRA1*, and the associated receptor *GABARAPL1*. In addition, in the pool of *GPHN* transcripts, we found an increased proportion of mRNAs containing retained intron 8 (visualized in Figure 4D), which reflects a reduction in the number of molecules encoding a complete functional protein structure. The three listed phenomena are highly likely to reduce the excitability of adenohypophyseal cells (all or selected lineages) to GABA molecules, increasing the independence of cell function from the availability of this neurotransmitter in the microenvironment.

Glutamate’s broad effects on the secretory activity of the AP cells include stimulation of FSH, LH and GH secretion and a rapid increase in PRL release via ionotropic receptors and inhibitory effects via metabotropic receptors [73,74,75]. Within EP samples, we found upregulation of the ionotropic glutamate receptor *GRIK2* gene expression (also called GluR6), which we further confirmed by the qPCR technique. At the same time, the lower concentration of *GRIK2* mRNA containing intron 11 retention was observed. Unfortunately, there is currently a lack of knowledge regarding the function of this receptor in the AP cells. Another important protein indirectly involved in glutamate reception is SLC1A2 (also referred to as EAAT2 or GLT-1). Berger and Hediger [76] noted the expression of the *SLC1A2* gene in a limited subset of the rat AP cells, simultaneously indicating the similarity of expression in brain astrocytes and speculating on the expression specificity limited to folliculo-stellate cells. Numerous research studies have shown that astroglial SLC1A2 transporters play a major role in maintaining physiological extracellular glutamate levels, and their expression and activity must be rigorously regulated due to their essential role in protecting neurons from excitotoxicity [77,78]. On the basis of cited similarity, we infer the presence of identical mechanism in the porcine AP, which was manifested by upregulation of *SLC1A2* expression levels in the EP group against ML samples. Increasing the number of SLC1A2 molecules, especially on folliculo-stellate cells, would affect the sealing of secretory cells from death during the peri-implantation period and would be part of the AP’s functional homeostasis preservation.

### 3.3. Intracellular Signal Transduction—Secondary Messengers

The pituitary cells integrate hormonal signals from the hypothalamus, intra-pituitary and peripheral glands mainly through G protein-coupled metabotropic receptors, which in turn trigger the release of secondary messenger molecules [79], such as Ca^2+^, cyclic adenosine monophosphate (cAMP), cyclic guanosine monophosphate (cGMP), diglyceride (DAG) and inositol trisphosphate (IP_3_). Functional meta-analyses showed significant effects of the transcriptomic phenomena observed in this study on the signaling pathways conducted by each of these molecules (Figures S2, S3, S9, S18 and S20), and on DAG and IP_3_ metabolic processes (Figure S12).

Activation of GnRH (in gonadotrophs) and TRH receptors (in lactotrophs and thyrotrophs) causes an intracellular increase of IP_3_ and DAG production, leading to the release of Ca^2+^ from cellular stores [80,81]. In this process, a key role is played by the PLCB4 and PLCG1 phospholipases [82,83]. Both genes are expressed in all rat pituitary cell lineages [83]. Comparing EP vs. ML groups, within the pool of *PLCB4* mRNAs, we detected an increase in the fraction containing the exon 2 long variant encoding the 5′ UTR fragment, and an increase of *PLCG1* transcripts incorporating intron 31. Involved in intracellular IP_3_ reception and Ca^2+^ efflux is the product of the *ITPR2* gene [84], for which we also identified an increase in the transcript fraction retaining intron 5, leading to increased production of RNA decays. Moreover, we identified the modifications of transcriptomic processes related to IP_3_ and DAG metabolism: *CDS1* (missense ASE within exon 10 resulting in Thr substitution with Pro), *DGKE* (downregulated DEG in EP vs. ML comparison), *DGKG* (upregulated exon 15 skipping), *ISYNA1* (downregulated DEG, expression verified with qPCR), *MTMR7* (missense ASE within exon 15 resulting in Thr substitution with Lys), *PEMT* (downregulated exon 3 skipping) and *PI4K2B* (missense ASE within exon 10 resulting in Val substitution with Met; female corticotroph-specific gene expression [56]). Furthermore, in the *CAMKK1* gene, belonging to a Ca^2+^-triggered signaling cascade and involved in a multitude of cellular processes (proteins confirmed in the human APs [85]), the occurrence of a missense ASE event within exon 16 was noted, providing a predictable substitution of Lys with Glu. The complexity of the mechanisms involved in IP_3_ and DAG production, Ca^2+^ release and their signaling pathways, as well as the abundance of mRNA and associated transcriptional modifications during the peri-implantation period, makes it impossible to give a clear indication of the direction of the changes taking place. Rather, the plethora of observations made in this area illustrates the complexity of intracellular transcriptional mechanisms aiming to maintain the AP homeostasis.

On the other hand, CRH (in corticotropes) and GHRH (in somatotropes) receptor activation shows an increase in the intracellular cAMP production [86,87]. The protein encoded by the *PDE5A* gene is involved in the intracellular control of cGMP and cAMP concentrations by catalyzing their degradation [88]. In the *PDE5A* mRNA pool in the EP group, we found an enlargement in the retention of intron 11 relative to the ML group. This may affect the abundance of transcripts encoding the fully functional proteins, and thus deferred degradation of both cAMP and cGMP.

GNAL is one of the G proteins involved in the transduction of dopamine receptor-derived signals, and its activation increases intracellular cAMP levels [89,90]. Its presence has been confirmed in the human pituitary gland [91]. In this research arrangement, we identified an increase in *GNAL* gene expression and positively correlated *trans*-interactions of its mRNAs with six DELs. We predict that increasing the quantity of active GNAL proteins may lead to increased amplification of dopaminergic signaling, which would have an inhibitory effect on the secretory activity of lactotrophs [92].

### 3.4. Intracellular Signal Transduction—PI3K/AKT and MAPK Pathways

Roof and Gutierrez-Hartmann [93] provided evidence that the balance of Ras/ERK and PI3K/AKT signaling is required to maintain pituitary homeostasis against tumorigenesis. Our analyses of the RNA-Seq datasets indicate the occurrence of transcriptomic phenomena affecting multilevel mechanisms of MAPK regulation (Figure S6) and PI3K/AKT pathway (Figure S10) activity, also in changing physiological states, affecting AP functions.

The gene whose expression is affected by the greatest number of peri-transcriptional events is *POSTN*. During subsequent analyses of adenohypophyseal cell transcriptomes, in a comparison of EP vs. ML groups, we detected a downregulation of this gene expression, an increase in exon 17 skipping and a negative correlation of expression with the three DELs that can be bound by the *POSTN* transcripts. We confirmed an increase in the fraction of *POSTN* transcripts lacking exon 17 in EP samples, with a simultaneous decrease in gene expression level by PCR (Figure 4A and Figure 7B). POSTN is an extracellular matrix-secreted protein, which binds to integrin receptors on cell membranes and in turn activates the PI3K/AKT signaling pathway and STAT3 (described later) transcription factor [94,95]. POSTN protein production was previously confirmed in the human AP glands [96]. On the other hand, Labrèche [97] demonstrated the presence of proteins in the bovine pituitary extract (hormone-rich proliferation-promoting supplement) acting as a natural repressor of *POSTN* expression in vitro and identified a mechanism of cross-stimulation/inhibition of this gene expression involving the PI3K/AKT pathway. We speculate that abundance of transcriptomic phenomena associated with the *POSTN* gene tending to suppress its expression may be a direct symptom of the multiplicity of changes in signaling pathways (in particular PI3K/AKT), modifying the intra-pituitary activity of the porcine cells during early stages of pregnancy.

This study revealed the presence of transcription-associated modifications within genes related with both the classically activated MAPK signaling pathway and the alternative transduction pathway involving JNK kinases. A number of publications indicate MAPK cascades activation by both GnRH and TRH [98,99]. The Ca^2+^ extracellular influx through L-type channels appears to be necessary for the activation of ERK kinases in the classical MAPK cascade [100]. *CACNA1D* gene encodes the pore-forming subunit of mentioned voltage-dependent Ca^2+^ channel type. Comparing the EP vs. ML groups, we detected a reduced expression level of this gene (confirmed by the qPCR technique) and an increase in the fraction of transcripts conserving intron 30. The predicted effect of these observations seems to be a reduction in the number of CACNA1D receptors in the membranes of the adenohypophyseal cells. In the long term, this may manifest itself in reduced Ca^2+^ uptake from the external environment. DUSP2 is involved in the inactivation of both ERK and JNK proteins [101]. During this study, we observed an increase of *DUSP2* gene expression, and a positively correlating mRNA–lncRNA *trans*-interaction with *MSTRG.14404.1*. We confirmed the expression profiles of both DEG and DEL by the qPCR technique. Moreover, in EP samples, we identified an increase of *MKNK1* mRNA fractions containing exon 2 skipping, promoting the expression of variants with a longer 5′ UTR fragment and yielding difficult-to-predict effects on the course and effect of translation. MKNK1 is activated by ERK, further leading to transcription factors activation [102]. Due to its multitude of functions, the PPP3CB calcineurin subunit plays an important role in the transduction of intracellular Ca^2+^-initiated signals [103]. Brinegar et al. [104] discovered that alternative splicing associated with murine *PPP3CB* exon 13 skipping/retaining can directly affect the activation and nuclear localization of NFATC transcription factors and/or affect the NFATC transcription targets’ expression. In the course of this study, within the *PPP3CB* gene, we identified two DAS events, classified as A5SS and RI, associated with the encapsulation of exon 13 (with 100% sequence identity to the mouse ortholog; alignment not shown) with the 3′ UTR sequence. As a result, there is an increase in the fraction of mRNA carrying exon 13 information untranslated into protein in EP samples relative to the control, likely affecting lower activation of NFATC proteins. MAPK10 (in literature often named JNK3) is one of the major early response kinases of the MAPK signaling pathway, whose activation requires the release of Ca^2+^ from intracellular stores [100]. Activated MAPK10 molecules can activate elements of the AP-1 transcription factor complex, such as JUN (C-Jun). In the EP group, we identified increased retention of introns 7 through 10 within *MAPK10* transcripts, which results in increased production of RNA decays and affects lower JUN activation. The reported data quite clearly indicate the inhibition of signal transduction by the MAPK cascades in the adenohypophyseal cells during the EP gestation period compared to the control physiological period.

On the other hand, FOS (also referred to as c-Fos) is a member of the nuclear phosphoproteins that build the AP-1 complex, and its synthesis can be induced very rapidly and temporarily by diverse biological signals [105,106]. AP-1 is known to regulate numerous processes including proliferation and apoptosis. Literature data suggest that FOS may be the master switch that transforms short-term stimulation into long-term responses such as the normal cell growth and differentiation [107]. FOS appears to be one of the key transcription factors in the AP cells due to the cell-specific expression induced, i.e., by GnRH, CRH, TRH and estrogen actions [108,109,110,111], as well as direct involvement in *FSHB* and *LHB* expression [99]. Intron 1 plays a special role in *FOS* gene expression, within which a series of control elements have been identified that have a stimulatory effect [111,112], even in the promoter’s absence. Van Haasteren et al. [111], in studies conducted on the mouse AP cells, showed increased RNA transcription of FOS gene constructs containing intron 1 under the synergistic stimulation by Ca^2+^ and cAMP. Listerman et al. [113] noted that *FOS* transcription involves kinetic competition between factors responsible for mRNA elongation and co-transcriptional splicing of intron 1. In the EP samples, we found a decreased fraction of *FOS* transcripts containing retained intron 1 relative to the ML group (Figure 4B). The observed change may be a direct result of a decrease in the signal transduction via Ca^2+^- and cAMP-dependent pathways. This suggestion is supported by the high conservativity between *FOS* intron 1 sequences in the mammalian organisms, indicating that they have similar structural elements and thus perform similar regulatory functions. Pairwise sequence alignments of porcine *FOS* first intron show 79.8% sequences identity with the human ortholog and 73.0% identity with the murine sequence (alignments not shown). On the other hand, Vargas et al. [114] proposed a mechanism involving the presence of unspliced *FOS* pre-mRNA certain concentration in the nucleoplasm of cells, which upon receiving specific stimuli induces splicing into mRNA. This would result in the rapid production of the needed protein isoforms. As revealed in this study, the DAS event most likely leads to increased production of active FOS molecules within adenohypophyseal cells during early pregnancy, presumably partially affecting the alteration of the examined transcriptomes structures.

### 3.5. IL6 Signal Reception Modifications

Unfortunately, the experiments described above have only a few intersections with the research of Zmijewska et al. [28], which also focuses on transcriptomic changes in the porcine adenohypophysis during early pregnancy. Three DEGs coincided with the results obtained in this microarray experiment: *CPT1A*—downregulated in both studies, *RBP4*—downregulated in both studies and *SYTL3*—upregulated in Zmijewska’s study and downregulated in this study. Despite the use of similar experimental material (both obtained on days 15–16 of pregnancy) and RNA isolation methods, the final result may be primarily affected by different control material (in our study, days 10–12 of the estrous cycle, the mid-luteal phase; in Zmijewska’s et al.’s study, days 15–16 of the cycle, the luteolytic phase) and different transcriptome profiling technologies. The advantage of our research model is a fully developed and functional corpus luteum both during the EP and ML periods [6]. In contrast, during luteolysis, the endocrine activity of the corpus luteum cells is dramatically inhibited as a result of progressive luteal cells’ apoptosis, affecting the hormonal milieu of the gilt organisms [6,115]. The advantage of the RNA-Seq relative to cDNA microarrays is the ability to distinguish different transcriptional variants (including alternative splicing) and allelic imbalance expression [116]. RNA-Seq having a wider dynamic range of expression levels enables more accurate estimation of transcriptomic changes [116].

Nevertheless, Zmijewska et al. [28] reported a decrease of *IL6* gene expression in a similar study design. In our study, we did not observe direct modifications of transcriptional phenomena within this gene. However, we noted the effect of the peri-implantation period on the downregulation of the *IL6R* expression, and an increase in the expression of *IL6ST* gene allele fraction containing an Asp missense substitution with Glu within exon 13, encoding the transmembrane helix domain. The products of these two genes are essential for IL6 signal transduction. Briefly, IL6 molecules are initially bound by the IL6R (often referred to as IL-6Rα) to establish complexes that, upon attaching to the IL6ST (also called gp130) and homodimerization, lead to signal internalization [117]. The presence of *IL6R* mRNA and proteins was detected in the rat APs, and in the human gonadotropic cells [118], whereas *IL6R* and *IL6ST* mRNAs expression was demonstrated in the rat corticotropes [119]. According to Schwartz [120], the IL6 intra-pituitary action may represent one of the key aspects of endocrine-immune interactions. Moreover, the fact that IL6 stimulates AP secretion of LH, FSH, GH, PRL and ACTH appears to be highly relevant [121,122,123,124]. A major signal transduction pathway activated upon detection of a chemical signal by the IL6R–IL6ST complex is JAK/STAT (Figure S21) [117,119,125,126]. The literature data indicate IL6-dependent activation of STAT3 proteins, which further implicates the increase of *POMC* expression in the corticotropic cells [127,128]. What is extremely interesting, in the *STAT3* gene, is that we identified an RNA editing event modification within exon 23, described in the PRESDB database as ovarian-specific. Following this, the predicted reduction of the IL6R molecules abundance through gene downregulation and the likely IL6ST spatial structure alteration may lead to a reduction of the AP-forming cells’ sensitivity to IL6 and may explain further described *POMC* expression downregulation.

### 3.6. Mechanisms Influencing the Course of Transcription and Translation Processes

Functional analysis indicated changes of the transcriptomic phenomena in the porcine AP cells during the embryo implantation period, with substantial effects on the processes of mRNA production, processing and transport, such as ‘mRNA surveillance pathway’ (Figure S2), ‘Spliceosome’ (Figure S5) and ‘RNA transport’ (Figure S6). Similarly, the manufacturing, folding and degradation mechanisms of protein macromolecules modifications, such as ‘Protein processing in endoplasmic reticulum’ (Figure S4) and ‘Ubiquitin mediated proteolysis’ (Figure S17) were identified.

The first process that pre-mRNA molecules undergo, predominantly already during transcription in the cell nucleus, is ‘5′ capping’ [129]. Essential in this process is the methyltransferase complex, which includes the RNMT enzyme, composed of the N-terminal and catalytic domains. The N-terminal domain is immensely important during the recruitment of protein complexes by transcription initiation sites on chromatin, thereby increasing the stability of the transcripts [130]. Within the two *RNMT* transcripts, we identified an ASE event manifested by SNV fractional imbalances within exon 1, resulting in an increased occurrence at 185 CDS position of T in favor of G (C > A on the sense) in the EP group relative to the control. The protein effect of this change is a missense substitution in the N-terminal domain of a hydrophobic Ala by a negatively charged Glu at position 62. The biological effect of the alteration observed within RNMT is difficult to predict; however, a similar ASE has been previously reported in the porcine hypothalamus [131] and has been linked to increased weights of selected muscles. This may induce spatial changes in the encoded enzyme molecules, influencing the gene expression regulation, and can point out the significance of the detected change across the hypothalamic–pituitary axes.

The post-transcriptional pre-mRNA processing step of great importance in the eukaryotic cells is RNA splicing, during which a ribonucleoprotein catalytic complex—spliceosome—catalyzes two transesterification reactions, in which an intron is initially excised and released as a lariat structure, and then adjacent exons are ligated [132]. The spliceosome is assembled on each intron and is mostly composed of small nuclear ribonucleoproteins (snRNPs)—*U1*, *U2* and *U4–6*—and more than 100 associating proteins [132,133]. In canonical splicing, *U1* is the first factor binding to the pre-mRNA and plays a crucial role in defining the location of the 5′ splice site based on the complementary base pairing of the 9 nt sequence at the 5′ end [134]. At subsequent steps, *U6* binds to the previously selected 5′ intron end, and via base pairing establishes complexes with *U4*, *U5* and *U2*, continuing the splicing course by forming a lariat-shaped intron, which is subsequently cleaved from the transcript [135]. Analyzing the RNA-Seq data, we discovered differences in the expression of four snRNAs—*U1.46*, *U1.52*, *U6.491* and *U6.856* (the numbers after the dot were introduced by the Ensembl for variant identification), with the expression-level downregulation of the first one and upregulation of the three remaining ones. Each of these snRNPs showed *trans*-actions with 17, 4, 30 and 20 DELs, respectively, based on RNA–RNA-binding strengths and expression profile correlations (46 positive and 25 negative). The altering effect of *U1* molecules on modifications to the spliceosome formation and constitutive splicing processes resulting from differences in the expression of distinct variants has been repeatedly presented [134,136]. Until recently, *U6* was considered an internal control when analyzing noncoding RNAs; however, it is now thought that the expression of genes encoding it depends on both physiological and pathological factors [137,138]. Moreover, recent reports have shown the existence of a phenomenon in which *U1* molecules can recruit lncRNAs with potential *U1*-binding sites to chromatin [139,140]. The significance of this mechanism is so far poorly understood, but it appears that such binding is essential for the function of lncRNAs in the nucleus, such as the regulation of chromatin structure and RNA transcription [139,140]. There is a gap in knowledge regarding *U6*–lncRNA interactions. Of the seven DELs molecules validated by qPCR in this work, five revealed *trans*-action with at least one of the mentioned snRNPs: *ENSSSCT00000074581*, *ENSSSCT00000076568*, *MSTRG.18944.1*, *MSTRG.20172.2* and *MSTRG.27546.1*.

We also found DAS events within a range of protein-coding genes that may encode the structural components of spliceosomes. Within the *U2AF1L4* gene (also described as *U2af26*), a pre-mRNA splicing factor, we identified an increase of transcript variants carrying exon 5 inclusion (Figure 4C), encoding a fragment of the RNA recognition motif (RRM) protein domain, in EP samples. Goldammer et al. [141] presented the mechanism of oscillatory *U2AF1L4* alternative splicing in mice, involving an enhancer within the aforementioned exon that is involved in the cyclic changes of different transcript variants’ production. Moreover, in the *SRSF7*-derived transcripts pool whose proteins bind to this enhancer, and are required for oscillating *U2AF1L4* alternative splicing [141], we identified the increase of variants carrying retained intron 3 in the EP group, providing the predicted effect on decreased production of active protein macromolecules. Other factors in which DASs were observed in EP relative to ML samples are *AQR* (SE—increased inclusion of exon 2 containing a 5′ UTR fragment), *HNRNPA3* (RI—promotion of increased production of variants possessing a shorter 3′ UTR), *PRPF3* (RI—promotion of increased production of variants possessing a longer 5′ UTR), *RBMX* (RI—reduced production of variants having a shorter 3′ UTR), *SRSF4* (RI—promotion of deletion of intron 3 from transcripts and production of functional proteins) and *SRSF5* (RI and A3SS relating to the same fragment—promotion of RNA decay expression). Most of the proteins produced by the listed genes have the valence to bind RNA molecules [133]. The exception is PRPF3, which forms the precatalytic spliceosome complex by binding, i.e., to *U6* snRNA. Unfortunately, the effects of the presented DAS events on spliceosome function have been poorly described in the literature. Given that they are all involved in the constitutive and/or alternative mRNA splicing processes, in our opinion, they may be largely responsible for changes in the transcriptional profiles of the AP cells during early pregnancy and may be an important factor regulating the multitude of results obtained in this study.

An important protein involved in the alternative splicing mechanism appears to be CELF4. According to the literature, it is involved in the processes of exon skipping and inclusion and retention of introns, influencing more broadly the stability and localization of mRNA and the course of the translation process [142,143]. Several studies indicate that *CELF4* gene expression is restricted to the central nervous system and even to the brain-building structures [144]. However, data on the expression of this gene within the AP are lacking. During this research dataset analysis, the series of DAS events were observed within the *CELF4* gene, involving the promotion of transcript variants lacking 12 exon expression in the EP group relative to controls. The effect of this event is the modification of the RRM3 domain sequence involved in the binding of specific RNA sequences [142]. In practice, it may alter the binding specificity of the created CELF4 molecules towards particular mRNAs. We positively validated the observed modification using the PCR technique. Within the *CELF4* gene, we also noted two MXE cases favoring the production of transcript variants containing exon 10 and lacking exon 11, which may have similar effects on protein conformation to those described above, and an A5SS event, which may be related to the length of the 5′ UTR. Wagnon et al. [143] using the mouse brain, RNA–protein crosslinking techniques, CELF4 complexes’ immunoprecipitation and the resulting nucleic acids’ high-throughput sequencing, created the database of mRNA targets for the described protein. In this pool, they identified 2000 targets in the expression of which alternative splicing induced by CELF4 would be most relevant. Of the narrowed dataset, 83 genes intersected with the DASs obtained in this experiment (marked in Table S4). Based on this, we conclude that the aforementioned changes in alternative splicing of *CELF4* transcripts directly affected the occurrence of DAS events in *CACNA1D*, *FOS*, *GRIK2*, *ITGAV*, *PPP3CB*, *UBA6* and other mRNAs, which may be connected with the adaptation of the AP cell activity to changing physiological conditions.

The culminating stage of mRNA molecules’ maturation is the polyadenylation of the 3′ end. A key protein involved in the polyadenylated RNA metabolism is PABPN1, whose function is the stimulation of poly(A) tail synthesis by increasing the poly(A) polymerase processivity and also controlling the tail length [145]. Moreover, it has been shown that PABPN1 is involved in specific gene expression regulation, by controlling polyadenylation signal recognition and affecting the 3′ UTR length, which has been termed alternative polyadenylation [146,147]. Among the *PABPN1* gene-derived mRNAs, we detected the DAS event involving an increase in the fraction of transcript variants retaining the last intron in EP samples compared to ML. Bergeron et al. [148] described the autoregulatory mechanism, observed in the human cells involving post-transcriptional splicing of *PABPN1* transcripts 3′-terminal intron, which is directly controlled by PABPN1 proteins. This homeostatic control operates by the negative feedback loop and is activated when active PABPN1 proteins are abundant in the nucleus, leading to the increase of its mRNA non-fully spliced molecules’ fraction, which promotes their decay by the exosome, further reducing the concentration of the PABPN1 molecules in the cells. The observed phenomenon reflects the increase of primarily protein-coding gene transcriptional activity during the EP period. This may cause the PABPN1 protein overproduction, which triggers the self-control process and peculiarly marks the transcriptional and translational processes intensification in the AP cells during peri-implantation period in relation to the ML phase.

The analyzed RNA-Seq datasets enabled the distinction of transcriptional mechanisms affecting protein maturation and degradation. UBA6 is E1 enzyme that initiates ubiquitinated protein degradation by activating ubiquitin and attaching it with a high-energy bond, allowing for the next steps of the process to occur [149]. Comparing EP and ML samples, we found the increase of *UBA6* transcripts containing intron 2 retention, most likely affecting the increased production of degraded RNA decays. Moreover, we detected the appearance of transcriptomic phenomena modifications in the following genes involved in the ligation of the ubiquitin molecules with the target protein: *CUL4A* (DAS related with a downregulated intron 18 retention), *CUL7* (missense ASE within exon 5 resulting in Ile substitution with Ser within ‘Armadillo-type fold’ domain), *MGRN1* (DAS involving enhanced retention of intron 2) and *PPIL2* (DAS relying on exon 3 skipping upregulation). The listed changes in the transcription-associated processes seem to imply the suppressive effect of the peri-implantation period on ubiquitin-dependent degradation of protein molecules in AP cells of gilts.

### 3.7. Predicted Secretory Effects

Proopiomelanocortin (POMC) is a complex polypeptide precursor that contains several biologically active proteins in its structure. In the AP, *POMC* gene is expressed primarily in the corticotrophs [150]. The precursor molecules produced by this cell lineage are proteolytically cleaved to ACTH. In the present study, we identified the downregulation of *POMC* gene expression in the EP vs. ML comparison, as well as an expression profile correlation with two DELs—*MSTRG.17573.1* and *ENSSSCT00000074581* (positive and negative, respectively)—associated with the adjustment of the spatial structures, including both mRNA and protein, and the occurrence of ASE in the 3′ UTR region of the three encoded transcript variants. Changes in *POMC* and *ENSSSCT00000074581* expression levels predicted by RNA-Seq data analysis were confirmed using qPCR. A direct cause of the decreased *POMC* gene expression and the occurrence of mechanisms likely to reduce POMC peptide levels in the AP cells may be the transcriptomic phenomena described above affecting the quantity and quality of *IL6R*, *IL6ST* and *STAT3* mRNAs. Many available reports indicate that, both in vivo and in vitro, IL6 stimulates the production and secretion of ACTH by the pituitary gland in humans and animals [151,152,153]. The multitude of transcription-related phenomena indicates a strong influence of the early gestation period on the inhibition of POMC molecule production, and thus also on the ACTH secretion reduction by the AP. The above observations supplement the mechanism postulated by Brunton et al. [154] and are supported by the observations of Nepomnaschy et al. [155], concerning the decrease in the HPA axis activity during early pregnancy, manifested by reduction of adrenal steroid secretion. Such a mechanism would be important in the pre-implantation period to prevent maternal stress-induced pregnancy loss [156].

## 4. Materials and Methods

### 4.1. Experimental Animals and Samples Collection

The research was carried out on cross-bred (Large White × Polish Landrace) mature gilts at age of 7–8 months and the weight of 130–140 kg. Ten animals (n = 5 per group) were used to examine transcriptomic differences in AP between early pregnancy and the estrous cycle. Considering that, in early pregnancy, pituitary and ovarian secretory activity is comparable to that observed on day 10–12 of the estrous cycle, pituitaries harvested during the mid-luteal phase of the estrous cycle were treated as a control group for samples collected from pregnant gilts [5,157,158]. Mid-luteal gilts were observed daily for estrus behavior in the presence of boar. The day of onset of the second estrus was designated as day 0 of the estrous cycle. The phase of the estrous cycle was additionally confirmed based on ovarian morphology according to Akins and Morrissette [159].

**Figure 8:**
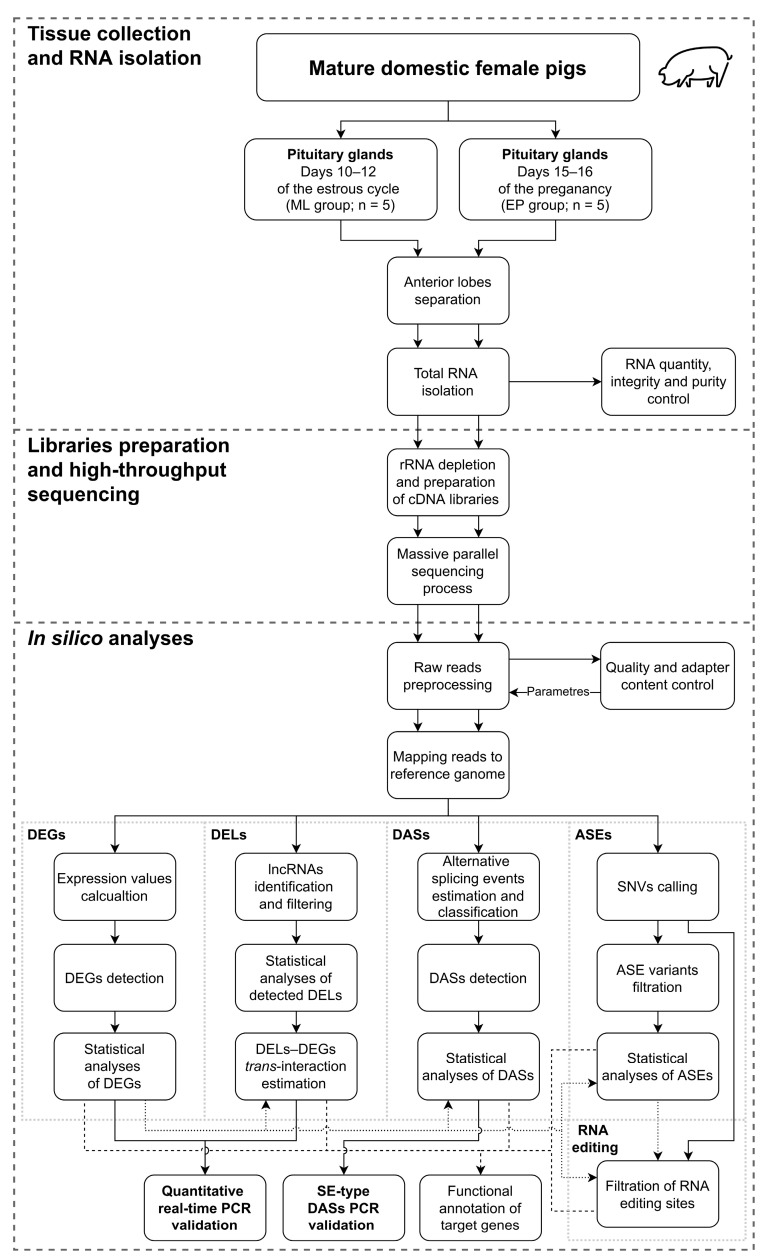
A stage-by-stage flowchart of the experiment performed in the scope of this article. Abbreviations: ASEs—allele-specific expression variants, cDNA—complementary DNA, DASs—differential alternative splicing events, DEGs—differentially expressed genes, DELs—differentially expressed lncRNAs, EP—the embryo implantation period of pregnancy, lncRNAs—long noncoding RNAs, ML—the mid-luteal phase of the estrous cycle, PCR—polymerase chain reaction, rRNA—ribosomal RNA, SE—skipping exon, SNVs—single nucleotide variants.

The insemination of five gilts was performed on days 1–2 of the estrous cycle. Additionally, pregnancy was confirmed by the presence of morphologically normal conceptuses in both uterine horns, and the day of pregnancy was also confirmed by the size and morphology of embryos according to Anderson [160]. For the pituitary samples intended for further high-throughput transcriptome sequencing, qPCR and PCR were collected post-mortem from the same animals and at the same time immediately after humanitarian slaughter. Pituitaries were placed in Dulbecco’s modified Eagle’s medium (DMEM; Sigma-Aldrich, St. Louis, MO, USA) supplemented with 100 IU/mL penicillin (Sigma-Aldrich, St. Louis, MO, USA) and 100 µg/mL streptomycin (Sigma-Aldrich, St. Louis, MO, USA) and transported to the laboratory on ice. The pituitary glands were dissected and divided into AP and NP, and further research was continued only on APs. The anterior lobes were frozen in liquid nitrogen and stored at −80 °C until processing for RNA analysis. The whole procedure of this research is summarized in Figure 8.

### 4.2. RNA Isolation, Library Preparation and High-Throughput Sequencing Procedure

Total RNA was extracted from all AP samples using RNeasy Mini Kit (Qiagen, Germantown, MD, USA) with DNase contained in RNase-free DNase Kit (Qiagen, Germantown, MD, USA), following the manufacturer’s protocol. The RNA purity (A_260_/A_280_) and quantity (A_260_) were estimated spectrophotometrically using Infinite M200 Pro (Tecan, Männedorf, Switzerland). The total RNA integrity was validated using Bioanalyzer 2100 (Agilent Technology, St. Clara, CA, USA). The RNA integrity number between 8 and 10 qualified the isolated RNA for further processing, such as RNA-Seq analysis, PCR and qPCR validations. Isolated RNA probes were stored at −80 °C for sequencing library preparation.

According to Shen’s et al. [161] recommendations, the sequencing libraries were prepared by purifying RNA solution from rRNA with the use of Ribo-Zero rRNA Removal Kit (Illumina, San Diego, CA, USA). The rRNA-depleted RNA samples were used to prepare strand-specific RNA-Seq libraries using the TruSeq Stranded mRNA Library Prep Kit (Illumina, San Diego, CA, USA), according to the manufacturer’s procedure. Briefly, long RNA molecules were fragmented, and based on them, double-stranded cDNA molecules were synthesized by replacing dTTPs with dUTPs in reaction solution during the synthesis of the cDNA second strand. The resulting double-stranded cDNA fragments were subjected to the end repair and A-tailing processes. Finally, specific adaptors were ligated into the obtained cDNA fragments. The PCR amplifications were performed to enrich the cDNA libraries. The high-throughput sequencing of obtained libraries was performed using the NovaSeq 6000 platform (Illumina, San Diego, CA, USA) to generate 2 × 150 nt paired-end reads with an assumed minimum sequencing depth of 70 million reads per sample.

### 4.3. Bioinformatic Analyses

The mRNAs levels and transcription-regulatory mechanisms, such as lncRNAs production, alternative splicing of transcripts and promoting the production of transcripts encoded by one of the alleles available in the genetic material, were analyzed to investigate the effect of the early gestation period milieu on porcine AP cells. The in silico analyses were mainly performed according to Paukszto et al. [162] and Makowczenko et al. [163].

#### 4.3.1. Raw Reads Pre-Processing, Mapping to a Reference Genome and Differentially Expressed Genes Processing

The pre-processing of reads, mapping against the genome and estimation of transcript and gene expression levels were performed according to Paukszto et al. [162]. Generated during the sequencing process raw reads were quality controlled using FastQC software ver. 0.11.9 [164]. Adapters and low-quality fragments of raw reads (average *Q*_Phred_ score < 30, *Q*_Phred_ score at 5′ and 3′ ends <20) were trimmed out and reads were clipped to equal lengths of 120 nt (essential for the rMATS software ver. 4.1.0) using the Trimmomatic tool ver. 0.40 [165]. Processed reads were re-checked for quality and adapter content with FastQC. The resulting read sets of the analyzed samples were mapped to a reference genome *S. scrofa* ver. 11.1.104 obtained from the Ensembl database [31] using STAR software ver. 2.7.10a [166] with the parameters suggested by Jakobi [167]. The annotation and estimation of the transcripts and genes expression levels, and detection of novel TARs were performed using the StringTie tool ver. 2.2.0 [168], with interpreting strand-specific sequencing the ‘fr—firststrand’ parameter was activated. Counts per transcript and gene were calculated using the *prepDE* Python script released by the author of StringTie [169]. The DEGs analysis was performed within the R environment ver. 4.1.1 [170], using the DESeq2 tool ver. 1.34.0 [171], based on a negative binomial generalized linear model with assumed cut-offs: adjusted *p*-value < 0.05 and absolute value of log_2_(FC) ≥ 0.58. To estimate the data repeatability within the EP and ML groups, PCA and cluster analysis, based on the Euclidean distances and Pearson correlation measurements, were performed within the R environment ver. 4.1.1 [170].

#### 4.3.2. Long Noncoding RNA Analyses

Identification of lncRNAs in the porcine AP cells was performed according to Shen et al.’s [161] multi-stage procedure with modifications [162,163]. Initially, at the filtering stage, t low expression, protein-coding, single-exon and too short transcripts (length < 200 nt) were excluded from the processed dataset. Subsequently, the surviving transcripts were screened for coding potential using five tools: CNCI ver. 2.0 [32], CPAT ver. 1.2.4 [33], CPC2 ver. 2.0.1 [34], FEELnc [35] and PLEK ver. 0.2 [36]. In parallel, the sequences of survived transcripts were aligned to protein domains records of the Pfam database [37] using the “hidden Markov model”-based HMMER tool ver. 3.3.2 [38] and to the Rfam database ver. 14.4 [39] using the Infernal cmscan tool ver. 1.1.3 [40]. Transcripts lacking coding potential according to the results of three of the five cited programs (CPAT score < 0.78; CPC2 score < 0; Feelnc score < 0.558; CNCI and PLEK index = “noncoding”), with low similarity to protein domains in the Pfam database (e-value > 10^−3^) and lacking alignments to the Rfam database records, were referred to in the subsequent analysis stages. To the newly identified lncRNA pool, known lncRNAs for the pig provided in the Ensembl database [31] were added. The expression differences of lncRNAs between the early pregnancy and the cycle AP samples were computed using the DESeq2 tool ver. 1.34.0 [172], with a cut-off of absolute value of log_2_(FC) ≥ 0.58 and adjusted *p*-value < 0.05.

The further analysis steps were performed to discover the relationships between the obtained DELs and DEGs using a high-throughput genomic data analysis toolkit—Bioconductor ver. 3.14 [173] within the R environment ver. 4.1.1 [170]. The relatively close localization of DEL and DEG on the same chromosome (up to 10,000 bp) was described as *cis*-interactions. The DEL–DEG pairs located in separate parts of the genome (*trans*-interactions) were characterized according to transcriptional profiles and on the binding affinity of lncRNAs and DEGs-encoded macromolecules (mRNA and protein). The predicted interdependence between transcriptional profiles of DELs and DEGs was examined using Pearson’s correlation coefficient. The lncRNA–mRNA binding strengths were computed as the normalized free energy (ndG) using the LncTar tool ver. 1.0 [41]. With the use of the lncPro tool ver. 1.0 [42], the probability of van der Waal’s interactions and hydrogen-bonding propensities between the secondary structures of the lncRNAs and potential protein targets were computed. Only those *trans*-interactions for which correlation analysis showed an absolute value of r > 0.9 and *p*-value < 0.05, and LncTar showed an ndG < −0.1 or lncPro indicated a probability > 0.9 were included in further analysis of lncRNAs’ function and their effect on DEGs expression. The DEGs and DELs expression profiles and *trans*-actions were visualized with Circos Perl-based software ver. 0.69-9 [174].

#### 4.3.3. Differential Alternative Splicing Events Analysis

Alternative splicing events were predicted using the rMATS tool ver. 4.1.0 [43] based on the output files provided by StringTie. DASs between EP and ML porcine AP groups were statistically tested, and the inclusion-level difference for all splicing events was computed. Detected DASs were considered statistically significant with FDR <0.05 and the absolute value of inclusion level difference >0.1. All discovered DASs were classified into five categories by rMATS: A5SS, A3SS, MXE, RI and SE. All DASs with a distinction involved in the relevant physiological processes identified during the functional analyses were visualized using the Circos software ver. 0.69-9 [174] and rmats2sashimiplot Python tool ver. 2.0.4 [175].

#### 4.3.4. Single Nucleotide Variants’ Detection, Allele-Specific Expression Events’ Identification and RNA Editing Sites’ Analyses

The single nucleotide variants occurring within transcripts were detected by aligning processed reads to the Ensembl’s *S. scrofa* reference genome sequences ver. 11.1.104 [31] (supplemented with data on known single nucleotide polymorphisms), following the procedure described by Paukszto et al. [162]. Calling of SNVs and estimation of differences in inclusion frequencies between the EP and ML groups were performed using a multi-stage pipeline consisting of the Picard tool ver. 2.6.0 [176], rMATS-DVR software ver. 1.0.0 [44] and a tool considered as the gold standard—GATK ver. 3.6.0 [45]. The following GATK standard parameters were applied during the subsequent ASEs and RNA editing sites’ processing steps to screen out low-quality and disrupted SNVs: root mean square mapping quality >40, total depth of base coverage >10, quality by depth >2, rank sum test for relative positioning of reference versus alternative alleles within reads >−8 and mapping quality rank sum >−12.5. Surviving SNVs were subjected to position-related analysis within filtered genes. SNVs located in the following regions were removed from the dataset: 5 bp range from the exon–intron junction within 50 bp up- and downstream range from paralog sequences, within the bidirectional genes and within simple sequence repeat regions (SSR; provided by the genome-wide microsatellite analyzing tool—GMATo ver. 1.2 [177]). Pairs or triplets of SNVs located in close proximity (2 within 45 bp or 3 within 35 bp) were also filtered out. Subsequently, SNVs with AAF >0 within at least half of the RNA-Seq samples were selected for further analyses.

Differences in the allelic expression of the discovered SNVs between the EP and ML groups were treated as statistically significant at the absolute value of ∆AAF > 0.1 and FDR < 0.001. The allelic imbalance ratio of survived ASEs was confirmed using the goodness-of-fit ꭕ^2^ test with a significance cut-off level of *p*-value < 0.05. The identified ASEs’ location within CDS regions and the SNVs’ effect on the specific proteins transcription process were determined using the Ensembl VEP tool [47]. Additionally, using the coverage ratio of the reference allele variant relative to an alternative variant, all ASEs were assigned to three categories: ‘true heterozygote’ when the ratio was close to 1:1, or ‘HeteroRef’/’HeteroAlt’ when the ratio indicated higher expression of one of the alleles.

To identify potential RNA editing sites, the filtration procedure was extended with additional steps, as suggested by Paukszto et al. [162]. From the dataset of SNVs surviving the series of described filtering steps (related to the quality parameters of the variants, their location and expression characteristics), only the canonical adenosine to inosine (A-to-I) and cytidine to uridine (C-to-U) substitutions without known Ensembl single nucleotide polymorphism ID (*rs* number in *S. scrofa* genome ver. 11.1.104) were selected for further analyses. SNVs with AAF >0.7 in any sample were filtered out according to the assumption that an RNA editing efficiency of 70% is improbable. Only SNVs, occurring along PRE-1 SINE regions within the porcine genome [178], were selected for final RNA editing dataset with the use of BEDTools software ver. 2.30.0 [179]. RNA editing events that did not reach the ‘FDR < 0.05’ and ‘absolute value of ∆AAF > 0.1’ premises were excluded from the dataset. The resulting A-to-I and C-to-U differential RNA editing events were compared with the records within the PRESDB database [48].

#### 4.3.5. Functional Annotation of Target Genes

Functional analysis was performed according to the procedure previously described by Makowczenko et al. [163]. Functional annotating processes, which consisted of gene ontology analyses and gene enrichment of signaling and metabolic pathways, were carried out using the KOBAS tool ver. 3.0 [49], screening three databases: GO [50,51], The Reactome Knowledgebase [52] and KEGG [53]. Annotation results were acquired separately for each of the phenomena analyzed—DEGs, DELs (based on *trans*-interactions with DEGs), DASs, ASEs and differential RNA editing events (for the latter three by occurrence within genes). All functional analyses were performed using Fisher’s exact test with Benjamini and Hochberg correction (FDR < 0.05). In order to combine and visualize the annotating results, all data from the KEGG databases were combined into clusters, and statistical significance values were recalculated for the regrouped data (FDR < 0.001). Molecular network maps including results for all transcriptomic phenomena examined in this manuscript were generated using the Pathview R package ver. 1.30.1 [180]. The DEGs-based GO output, considering expression profiles, was plotted with the GOplot R package ver. 1.0.2 [181].

### 4.4. Quantitative Real-Time PCR and PCR Validations

In order to validate the observed modifications of DEGs and DELs’ expression profiles, the qPCR technique was applied. The SE-classified DAS events were validated using PCR. The 1 µg of each RNA sample used in the RNA-Seq was reverse-transcribed to cDNA, yielding a total volume of 20 µL of each mixture, using the Omniscript RT Kit (Qiagen, Germantown, MD, USA) and 0.5 µg of oligo(dT)15 primers (Roche, Penzberg, Germany). The reverse transcription reaction was carried out at 37 °C for 1 h and was terminated by incubation at 93 °C for 5 min. Primer sequences for selected target genes (*CACNA1D*, *DUSP2*, *GRIK2*, *ISYNA1*, *NELFCD*, *POMC* and *VGF*), lncRNAs (*ENSSSCT00000074581*, *ENSSSCT00000076568*, *MSTRG.14404.1*, *MSTRG.18944.1*, *MSTRG.20172.2*, *MSTRG.27546.1* and *MSTRG.32275.1*) and SE events within genes (*CELF4* and *POSTN*) were developed using Primer Express software 3 (Applied Biosystems, Waltham, MA, USA). The primer sequences of selected references and targets are listed in Table 3.

The qPCRs were performed using the AriaMx Real-Time PCR System (Agilent Technology, St. Clara, CA, USA). The constitutively expressed *GAPDH* and *PPIA* genes were used as reference. Rection mixtures with a final volume of 21 µL consisted of 30 ng prepared cDNA, 200 nM of forward and reverse primers, 12.5 µL of the RT HS-PCR Mix SYBR (A&A Biotechnology, Gdansk, Poland), 0.24 uL of the ROX dye (A&A Biotechnology, Gdansk, Poland) and RNase-free water. The qPCRs were performed under the following conditions: preliminary cDNA denaturation and enzymes activation at 95 °C for 3 min, 40 cycles of denaturation at 95 °C for 30 s, annealing for 1 min and elongation at 72 °C for 30 s. The annealing temperatures for subsequent primers were as follows: 59 °C—*GAPHD*; 60 °C—*PPIA*, *ENSSSCT00000074581* and *MSTRG.14404.1*; 62 °C—*VGF*, *DUSP2*, *MSTRG.20172.2* and *MSTRG.18944.1*; 64 °C—*GRIK2*, *MSTRG.27546.1*, *ENSSSCT00000076568* and *MSTRG.32275.1*; and 66 °C—*NELFCD*, *POMC*, *CACNA1D* and *ISYNA1*. The qPCRs were performed in technical duplicate for each sample. Negative controls were prepared by substituting cDNA with water. Amplification specificity was tested at the end of reactions by melting curve analysis. The relative expression levels of the selected target genes and lncRNAs were calculated using the 2^−ΔΔCT^ method and normalized using the geometrical means of the reference genes’ expression in accordance with Livak and Schmittgen [184]. The Student’s *t*-test was applied to statistically process the qPCRs results, with a significant *p*-value threshold < 0.05. The calculations were carried out within the R environment ver. 4.1.1 [170], and results were visualized as log_2_(FC) ± confidence interval.

The PCRs were performed using Labcycler 48s (Syngen Biotech, Wroclaw, Poland). Rection mixtures, in a final volume of 25 µL, contained 30 ng of cDNA, 200 nM of forward and reverse primers, 12.5 μL of StartWarm HS-PCR Mix (A&A Biotechnology, Gdansk, Poland) and RNase-free water. The PCRs were performed following the procedure: preliminary activation at 95 °C for 3 min, 40 cycles of denaturation at 95 °C for 30 s, annealing at 60 °C for 1 min, elongation at 72 °C for 45 s and final extension at 72 °C for 7 min. The PCRs products were analyzed using 1.5% agarose gels containing Midori Green Advance (Nippon Genetics Europe, Duren, Germany).

## 5. Conclusions

This publication provides, for the first time, detailed insight into the subtle transcriptomic processes taking place in the anterior pituitary lobe cells of the domestic pig during the peri-implantation period of pregnancy, such as mRNA and lncRNA expression, alternative splicing of transcripts, allele-specific expression and RNA editing processes. The high-throughput analyses results presented in this study indicate the presence of sophisticated transcriptional mechanisms in the adenohypophyseal cells that probably have homeostatic functions and are associated with the maintenance of secretory activity of the gland. The observed self-controlling transcriptomic modifications seem to affect such physiological processes as signal reception from higher and lower branches of the HPG axis (GnRH and estrogens); intracellular signal transduction mediated by Ca^2+^, cAMP, IP_3_ and DAG; modification of the PI3K/AKT pathway activation; apparent MAPK pathway inhibition; and cross-regulation of the HPA axis through the IL6-dependent control of POMC expression. The results presented in this paper significantly expand our knowledge of the transcriptomic mechanisms involved in the regulation of reproduction in the domestic pig, but also, due to the high physiological similarity, may provide a basis for further studies on the human model.

## Figures and Tables

**Table 1:** Summary of the RNA-Seq results and the initial stages of in silico analyses—the raw reads processing and mapping them to the porcine reference genome (*S. scrofa* ver. 11.1.104). Numerical values are expressed in millions.

Physiological Condition	Mid-Luteal Phase (Days 10–12)					Early Pregnancy (Days 15–16)				
Samples	1_ML	2_ML	3_ML	4_ML	5_ML	1_EP	2_EP	3_EP	4_EP	5_EP
Raw reads	72.432	72.180	66.126	69.288	72.645	72.387	72.401	72.615	72.714	72.007
Processed reads	58.689	57.916	52.628	51.839	57.544	53.791	57.401	55.282	63.397	52.511
Mapped reads	56.773	55.304	50.692	50.814	55.763	53.031	56.484	52.697	61.209	51.657
Uniquely mapped reads	52.615	51.013	47.023	47.889	52.567	51.455	53.689	49.443	58.120	48.469
% of uniquely mapped reads	89.65%	88.08%	89.35%	92.38%	91.35%	95.66%	93.53%	89.44%	91.68%	92.30%
Multi-mapped reads	4.158	4.291	3.668	2.924	3.196	1.575	2.795	3.253	3.089	3.188
% of bases mapped to CDS	26.06%	24.76%	24.02%	18.71%	23.57%	18.94%	19.03%	22.94%	18.72%	19.80%
% of bases mapped to UTR	14.40%	13.14%	13.37%	10.63%	13.12%	12.68%	11.44%	12.65%	11.28%	11.07%
% of bases mapped to introns	33.71%	36.19%	37.59%	44.86%	37.73%	42.27%	46.44%	39.69%	46.73%	44.30%
% of bases mapped to intergenic	25.83%	25.92%	25.02%	25.80%	25.58%	26.11%	23.09%	24.72%	23.27%	24.83%

Abbreviations: CDS—coding DNA sequences, UTR—untranslated regions.

**Table 2:** Summary of KEGG pathways that are statistically implicated by identified DEGs; DELs associated with DEGs; and protein-coding genes associated with DASs, ASEs and differential RNA editing events in the porcine pituitary cells isolated during the embryo implantation period of pregnancy and the mid-luteal phase of the estrous cycle.

Pathway Name	Pathway ID	Input (Background) Number	FDR	Figure
Axon guidance	ssc04360	30 (155)	9.66 × 10^−7^	—
Focal adhesion	ssc04510	30 (155)	9.66 × 10^−7^	—
Phosphatidylinositol signaling system	ssc04070	22 (89)	1.48 × 10^−6^	Figure S2
Tight junction	ssc04530	28 (143)	1.55 × 10^−6^	—
cAMP signaling pathway	ssc04024	30 (175)	5.56 × 10^−6^	Figure S3
Protein processing in endoplasmic reticulum	ssc04141	26 (138)	6.76 × 10^−6^	Figure S4
Spliceosome	ssc03040	23 (113)	9.36 × 10^−6^	Figure S5
mTOR signaling pathway	ssc04150	25 (133)	1.02 × 10^−5^	—
MAPK signaling pathway	ssc04010	34 (233)	1.67 × 10^−5^	Figure S6
RNA transport	ssc03013	24 (129)	1.67 × 10^−5^	Figure S7
Thyroid hormone signaling pathway	ssc04919	22 (112)	2.03 × 10^−5^	—
Cholinergic synapse	ssc04725	18 (77)	2.03 × 10^−5^	Figure S8
Phospholipase D signaling pathway	ssc04072	24 (132)	2.03 × 10^−5^	Figure S9
Endocytosis	ssc04144	31 (206)	2.24 × 10^−5^	—
Regulation of actin cytoskeleton	ssc04810	28 (179)	3.22 × 10^−5^	—
Relaxin signaling pathway	ssc04926	21 (109)	3.33 × 10^−5^	—
PI3K-Akt signaling pathway	ssc04151	36 (271)	3.61 × 10^−5^	Figure S10
Glycerophospholipid metabolism	ssc00564	17 (74)	3.61 × 10^−5^	—
Glutamatergic synapse	ssc04724	18 (83)	3.64 × 10^−5^	Figure S11
Rap1 signaling pathway	ssc04015	27 (174)	4.59 × 10^−5^	—
Inositol phosphate metabolism	ssc00562	16 (68)	4.85 × 10^−5^	Figure S12
Dopaminergic synapse	ssc04728	19 (97)	6.46 × 10^−5^	Figure S13
GABAergic synapse	ssc04727	15 (65)	1.03 × 10^−4^	Figure S14
GnRH signaling pathway	ssc04912	16 (75)	1.18 × 10^−4^	Figure S15
Insulin resistance	ssc04931	18 (96)	1.50 × 10^−4^	—
Ras signaling pathway	ssc04014	27 (191)	1.50 × 10^−4^	—
Autophagy—animal	ssc04140	20 (116)	1.50 × 10^−4^	—
Prolactin signaling pathway	ssc04917	14 (60)	1.50 × 10^−4^	—
Purine metabolism	ssc00230	19 (107)	1.58 × 10^−4^	—
Circadian entrainment	ssc04713	14 (62)	1.87 × 10^−4^	—
HIF-1 signaling pathway	ssc04066	17 (91)	2.15 × 10^−4^	—
mRNA surveillance pathway	ssc03015	14 (65)	2.61 × 10^−4^	Figure S16
Neurotrophin signaling pathway	ssc04722	18 (103)	2.61 × 10^−4^	—
Inflammatory mediator regulation of TRP channels	ssc04750	16 (84)	2.74 × 10^−4^	—
Ubiquitin-mediated proteolysis	ssc04120	19 (114)	2.76 × 10^−4^	Figure S17
cGMP-PKG signaling pathway	ssc04022	21 (136)	2.90 × 10^−4^	Figure S18
Retrograde endocannabinoid signaling	ssc04723	19 (115)	2.90 × 10^−4^	—
Fc gamma R-mediated phagocytosis	ssc04666	15 (76)	2.99 × 10^−4^	—
Mitophagy—animal	ssc04137	13 (58)	2.99 × 10^−4^	—
Chemokine signaling pathway	ssc04062	22 (150)	3.51 × 10^−4^	—
ErbB signaling pathway	ssc04012	14 (70)	4.23 × 10^−4^	—
Estrogen signaling pathway	ssc04915	18 (111)	4.82 × 10^−4^	Figure S19
Oxytocin signaling pathway	ssc04921	19 (124)	5.91 × 10^−4^	—
ECM–receptor interaction	ssc04512	13 (64)	6.07 × 10^−4^	—
Sphingolipid signaling pathway	ssc04071	16 (94)	6.46 × 10^−4^	—
Amino sugar and nucleotide sugar metabolism	ssc00520	11 (48)	7.51 × 10^−4^	—
Apelin signaling pathway	ssc04371	18 (117)	7.71 × 10^−4^	—
Aminoacyl–tRNA biosynthesis	ssc00970	11 (49)	8.21 × 10^−4^	—
Fatty acid metabolism	ssc01212	11 (49)	8.21 × 10^−4^	—
Adherens junction	ssc04520	12 (59)	9.29 × 10^−4^	—

**Table 3:** Primers used for the PCR and qPCR validations of RNA-Seq data analyses results.

Gene Symbol	Type	Primers Sequences	Product Length	Reference
*CACNA1D*	DEG	F: CATCGCATCACTGCTGCTTC	185 bp	[The present study]
		R: TCACAGCGTTCCAGTCTTCC		
*DUSP2*	DEG	F: CATCCCTGTGGAGGACAACC	187 bp	[The present study]
		R: GGCCTCATCTAGACGCACTC		
*GRIK2*	DEG	F: TGGGAATGACCGGTTTGAGG	372 bp	[The present study]
		R: AGCACACAACTGACACCCAA		
*ISYNA1*	DEG	F: TACATCCCGGAGTTCATCGC	201 bp	[The present study]
		R: AGCAGTGTCATTGAGGCCAG		
*NELFCD*	DEG	F: ACACCTCTGACTTCGTGCAG	119 bp	[The present study]
		R: CGGGCAAAACCCACCTATGA		
*POMC*	DEG	F: AAAGTAACTTGCTGGCGTGC	363 bp	[The present study]
		R: CGTTGGGATACACCTTCACCG		
*VGF*	DEG	F: TGAAATCGCCCAGGTTGCC	164 bp	[The present study]
		R: AACATCCTTTGGCCCGATCA		
E*T00000074581	DEL	F: TTTTCCCAAAGGCAGGAGCA	414 bp	[The present study]
		R: TGATCTGTTTCGGCAGGCTT		
E*T00000076568	DEL	F: CAAGGCGGTCGTTAGGATCA	344 bp	[The present study]
		R: CACTAATGAAGGCGCTGCAC		
*MSTRG.14404.1*	DEL	F: GTGACATGTGTGGGACGGTA	110 bp	[The present study]
		R: CACGTCTTCCTGACAGCCTC		
*MSTRG.18944.1*	DEL	F: AGGAGTTCAAGGCCAACAGG	160 bp	[The present study]
		R: GTCCACGTACACCCCCTTTC		
*MSTRG.20172.2*	DEL	F: TATCCTGCACCGAGCAATGG	119 bp	[The present study]
		R: CAACCCAGACCATCCCATCC		
*MSTRG.27546.1*	DEL	F: GCTTTGTGTGGCCTGGACTA	333 bp	[The present study]
		R: TCCACATAGGCACAGAGGAGA		
*MSTRG.32275.1*	DEL	F: AGACAGTAAGCACACAGCGG	164 bp	[The present study]
		R: TGTGGAGTTGGATCATGGCG		
*CELF4*	DAS	F: TACCATCTGCCCCAGGAGTT	78 or 157 bp	[The present study]
		R: GTTGTCGAAGCTCACGAAGC		
*POSTN*	DAS	F: TCGACTAGTGGTGGCGAAAC	81 or 165 bp	[The present study]
		R: GGTGGCTTGTATCTTCCTCACA		
*GAPDH*	HKG	F: CCTTCATTGACCTCCACTACATGG	183 bp	[182]
		R: CCACAACATACGTAGCACCAGCATC		
*PPIA*	HKG	F: GCACTGGTGGCAAGTCCAT	71 bp	[183]
		R: AGGACCCGTATGCTTCAGGA		

Abbreviations: bp—base pairs; DAS—differential alternative splicing (skipping exon class); DEG—differentially expressed gene; DEL—differentially expressed long noncoding RNA; ‘E*’—‘ENSSSC’; F—forward primer; R—reverse primer.

## Data Availability

Details of the experimental procedures, raw reads and expression data obtained from all ML and EP RNA-Seq libraries were deposited in the European Nucleotide Archive database under accession number PRJEB56673 (https://www.ebi.ac.uk/ena/browser/view/PRJEB56673 (accessed on 16 October 2022)) and ArrayExpress Archive of Functional Genomics Data under accession number E-MTAB-12316 (https://www.ebi.ac.uk/arrayexpress/experiments/E-MTAB-12316 (accessed on 15 December 2022)).

## Supplementary Materials

The following supporting information can be downloaded at: https://www.mdpi.com/article/10.3390/ijms24065946/s1.

## Abbreviations

*AKAP11*	A-kinase anchoring protein 11
*ALDH3A2*	Aldehyde dehydrogenase 3 family member A2
*AQR*	Aquarius intron-binding spliceosomal factor
*ATRX*	ATRX chromatin remodeler
*BLTP2*	Bridge-like lipid transfer protein family member 2
*BMS1*	BMS1 ribosome biogenesis factor
*BNIP5*	BCL2-interacting protein 5
*BTAF1*	B-TFIID TATA box-binding protein-associated factor 1
*C16orf72*	Chromosome 16 open reading frame 72
*C2CD5*	C2 calcium-dependent domain containing 5
*CACNA1D*	Calcium voltage-gated channel subunit α1 D
*CAMKK1*	Calcium/calmodulin-dependent protein kinase kinase 1
*CAVIN2*	Caveolae-associated protein 2
*CCP110*	Centriolar coiled-coil protein 110
*CD47*	Surface antigen CD47
*CDS1*	CDP-diacylglycerol synthase 1
*CELF4*	CUGBP elav-like family member 4
*CNST*	Consortin, connexin-sorting protein
*CPT1A*	Carnitine palmitoyltransferase 1A
*CUL**	Cullin *(4A, 7)
*DERL3*	Derlin 3
*DGK*(E, G)*	Diacylglycerol kinase *(ε, γ)
*DISP1*	Dispatched RND transporter family member 1
*DMXL1*	Dmx like 1
*DNAJB4*	DnaJ heat shock protein family (Hsp40) member B4
*DUSP2*	Dual Specificity Phosphatase 2
*ELP1*	Elongator acetyltransferase complex subunit 1
*ESR2*	Estrogen receptor 2
*EXTL1*	Exostosin-likeglycosyltransferase 1
*FOS*	Fos proto-oncogene, AP-1 transcription factor subunit
*FSHB*	Follicle-stimulating hormone subunit β
*FZD3*	Frizzled class receptor 3
*GABARAPL1*	GABA type A receptor-associated protein-like1
*GABRA1*	GABA type A Receptor subunit α1
*GALP*	Galanin-like peptide
*GALT*	Galactose-1-phosphate uridylyltransferase
*GAPDH*	Glyceraldehyde-3-phosphate dehydrogenase
*GMPS*	Guanine monophosphate synthase
*GNAL*	G protein subunit α L
*GnRHR*	GnRH receptor
*GPHN*	Gephyrin
*GRIK2*	Glutamate ionotropic receptor kainate type subunit 2
*GTF2I*	General transcription factor IIi
*HNRNPA3*	Heterogeneous nuclear ribonucleoprotein A3
*IGSF1*	Immunoglobulin superfamily member 1
*IL6*	Interleukin 6
*IL6R*	Interleukin 6 receptor
*IL6ST*	Interleukin 6 cytokine family signal transducer
*ISYNA1*	Inositol-3-phosphate synthase 1
*ITGAV*	Integrin subunit alpha V
*ITPR2*	Inositol-1,4,5-trisphosphate receptor type 2
*LDHD*	Lactate dehydrogenase D
*LHB*	Luteinizing hormone subunit β
*LSR*	Lipolysis-stimulated lipoprotein receptor
*MAGI1*	Membrane-associated guanylate kinase, WW and PDZ domain containing 1
*MAP1A*	Microtubule-associated protein 1A
*MAPK10*	Mitogen-activated protein kinase 10
*MARCH8*	Membrane-associated ring-CH-type finger 8
*MIGA1*	Mitoguardin 1
*MKNK1*	MAPK-interacting serine/threonine kinase 1
*MMADHC*	Metabolism of cobalamin-associated D
*MGRN1*	Mahogunin ring finger 1
*MTMR7*	Myotubularin-related protein 7
*NDUFA10*	NADH:ubiquinone oxidoreductase subunit A10
*NELFCD*	Negative elongation factor complex member C/D
*NRN1*	Neuritin 1
*OCIAD2*	OCIA domain containing 2
*PABPN1*	Poly(A)-binding protein nuclear 1
*PARG*	Poly(ADP-ribose) glycohydrolase
*PDE5A*	Phosphodiesterase 5A
*PEMT*	Phosphatidylethanolamine N-methyltransferase
*PI4K2B*	Phosphatidylinositol 4-kinase type 2β
*PLC*(B4, G1)*	Phospholipase C *(β4, γ1)
*POMC*	Proopiomelanocortin
*POSTN*	Periostin
*PPIA*	Peptidylprolyl isomerase A
*PPIL2*	Peptidylprolyl isomerase-like2
*PPP3CB*	Protein phosphatase 3 catalytic subunit β
*PRPF3*	Pre-mRNA processing factor 3
*PTPRN2*	Protein tyrosine phosphatase receptor type N2
*RBMX*	RNA-binding motif protein X-linked
*RBP4*	Retinol-binding protein 4
*RNMT*	RNA guanine-7 methyltransferase
*ROBO1*	Roundabout guidance receptor 1
*RTN4*	Reticulon 4
*SEPTIN2*	Septin 2
*SRSF**	Serine- and arginine-rich splicing factor *(4, 5, 7)
*SIL1*	SIL1 nucleotide exchange factor
*SLC*(1A2, 9B2)*	Solute carrier family *(1 member 2, 9 member B2)
*STAM2*	Signal transducing adaptor molecule 2
*STAT3*	Signal transducer and activator of transcription 3
*SUCLA2*	Succinate-CoA ligase ADP-forming subunit β
*SYTL3*	Synaptotagmin-like3
*TAOK2*	TAO kinase 2
*TENM3*	Teneurin transmembrane protein 3
*TMED3*	Transmembrane P24 trafficking protein 3
*TMF1*	TATA element modulatory factor 1
*U*(1–6)*	U*(1, 2, 4, 5, 6) small nuclear RNA
*U2AF1L4*	U2 small nuclear RNA auxiliary factor 1-like4
*UBA6*	Ubiquitin-like modifier activating enzyme 6
*URGCP*	Upregulator of cell proliferation
*VGF*	VGF nerve growth factor inducible
*ZKSCAN5*	Zinc finger with KRAB and SCAN domains 5
*ZNF664*	Zinc-finger protein 664
