# Supplementary material for: Adaptation of the Porcine Pituitary Transcriptome, Spliceosome and Editome during Early Pregnancy
Source: Int J Mol Sci. 2023 Mar 21;24(6):5946. doi: 10.3390/ijms24065946 (PMC10053595; doi:10.3390/ijms24065946)
Supplement: Supplementary file 1 [file ijms-24-05946-s001.zip › ijms-2265868-supplementary captions.pdf]

International Journal of
Molecular Sciences

Table S1: Detailed DEGs analysis results.

Table S2: Detailed DELs analysis results.

Table S3: Detailed results of DEGs—DELs *trans*-interactions.

Table S4: Detailed DASs analysis results.

Table S5: Detailed ASEs analysis results.

Table S6: Detailed RNA editing analysis results.

Table S7: Results of GO and Reactome functional analysis of DEGs, DEGs acting with DELs, and genes incorporating DASs, ASEs and RNA editing events.

Figure S1: Venn diagrams visualizing the process of novel lncRNAs identification. (A) The relationship between the number of transcripts classified as 'noncoding' by each program: CNCI, CPAT, CPC2, Feelnc and PLEK. (B) The effect of aligning potential lncRNAs with Pfam and Rfam database records using HMMER and Infernal cmscan tools. The numbers contained inside the bold shapes represent the same dataset. The bold number expresses the final amount of novel lncRNAs identified.

Figure S2: Phosphatidylinositol signaling system with detected transcription-associated modifications mapped.

Figure S3: cAMP signaling pathway with detected transcription-associated modifications mapped.

Figure S4: Protein processing in endoplasmic reticulum with detected transcription-associated modifications mapped.

Figure S5: Spliceosome with detected transcription-associated modifications mapped.

Figure S6: MAPK signaling pathway with detected transcription-associated modifications mapped.

Figure S7: RNA transport with detected transcription-associated modifications mapped.

Figure S8: Cholinergic synapse with detected transcription-associated modifications mapped.

Figure S9: Phospholipase D signaling pathway with detected transcription-associated modifications mapped.

Figure S10: PI3K-Akt signaling pathway with detected transcription-associated modifications mapped.

Figure S11: Glutamatergic synapse with detected transcription-associated modifications mapped.

Figure S12: Inositol phosphate metabolism with detected transcription-associated modifications mapped.

Figure S13: Dopaminergic synapse with detected transcription-associated modifications mapped.

Figure S14: GABAergic synapse with detected transcription-associated modifications mapped.

Figure S15: GnRH signaling pathway with detected transcription-associated modifications mapped.

Figure S16: mRNA surveillance pathway with detected transcription-associated modifications mapped.

Figure S17: Ubiquitin mediated proteolysis with detected transcription-associated modifications mapped.

Figure S18: cGMP-PKG signaling pathway with detected transcription-associated modifications mapped.

Figure S19: Estrogen signaling pathway with detected transcription-associated modifications mapped.

Figure S20: Calcium signaling pathway with detected transcription-associated modifications mapped.

Figure S21: JAK/STAT signaling pathway with detected transcription-associated modifications mapped.
