# Supplementary figures and images for: Adaptation of the Porcine Pituitary Transcriptome, Spliceosome and Editome during Early Pregnancy
Source: Int J Mol Sci. 2023 Mar 21;24(6):5946. doi: 10.3390/ijms24065946 (PMC10053595; doi:10.3390/ijms24065946)

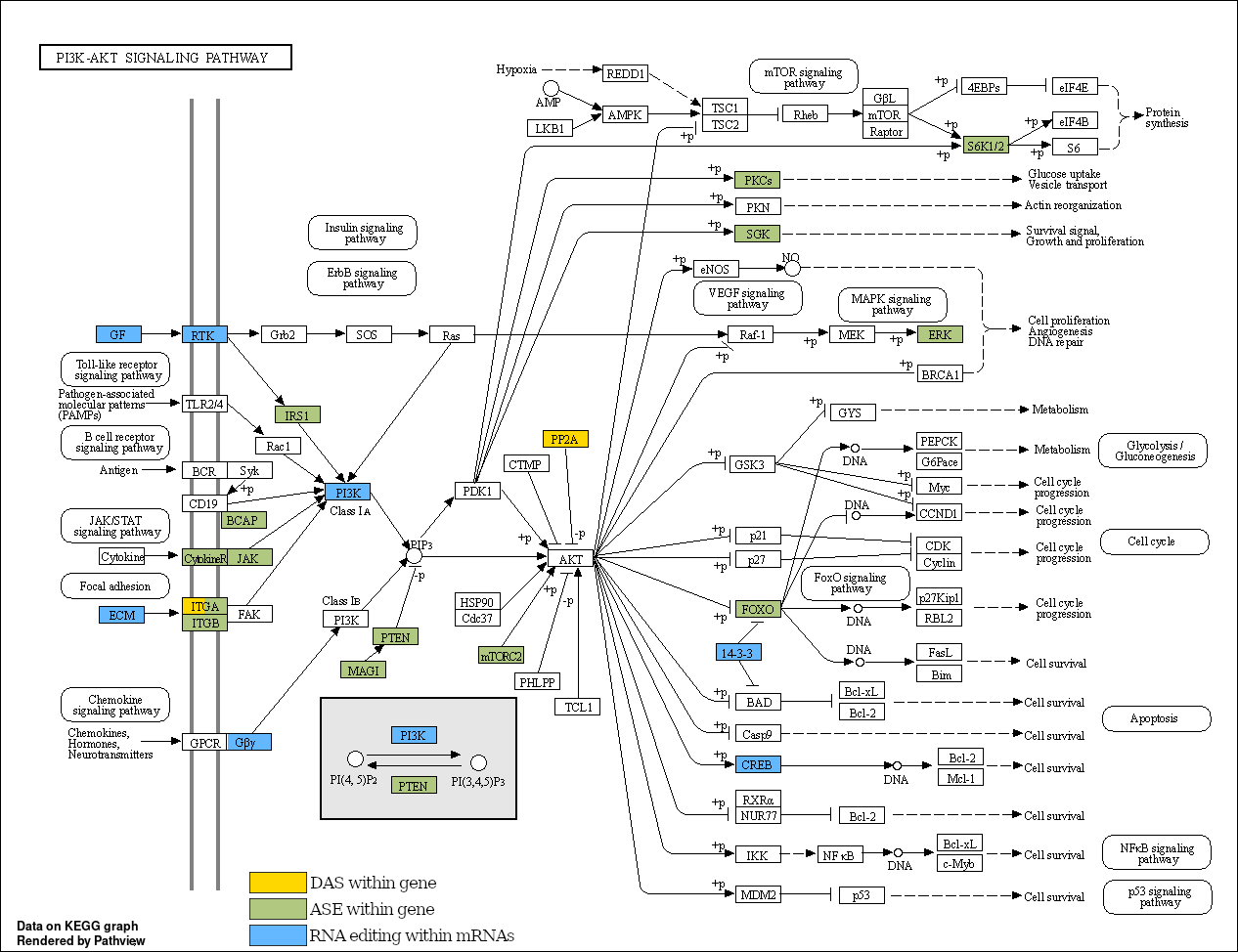

Supplement: Supplementary file 1 [file ijms-24-05946-s001.zip › Supplementary materials/Figure S10.png]

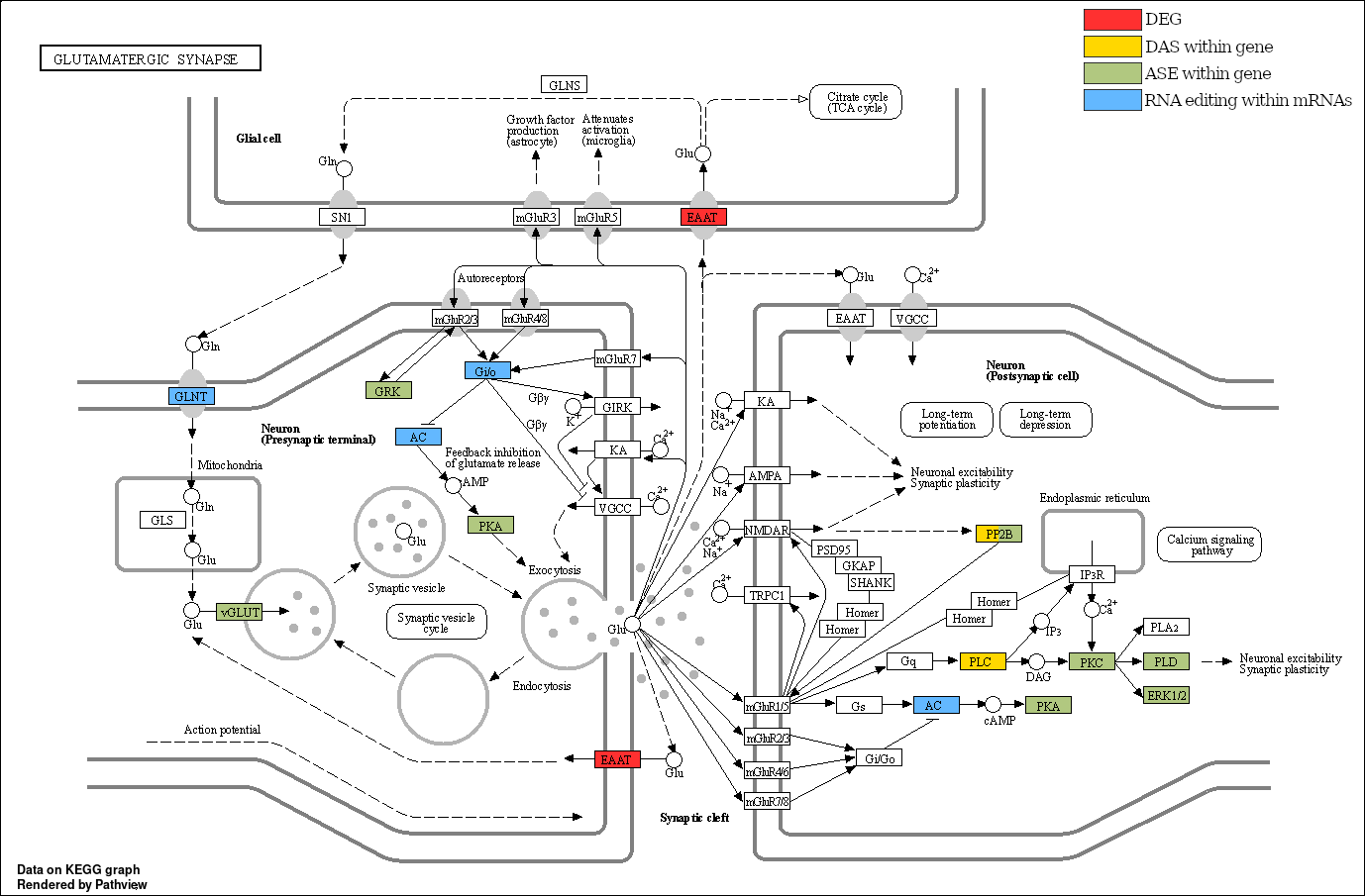

Supplement: Supplementary file 1 [file ijms-24-05946-s001.zip › Supplementary materials/Figure S11.png]

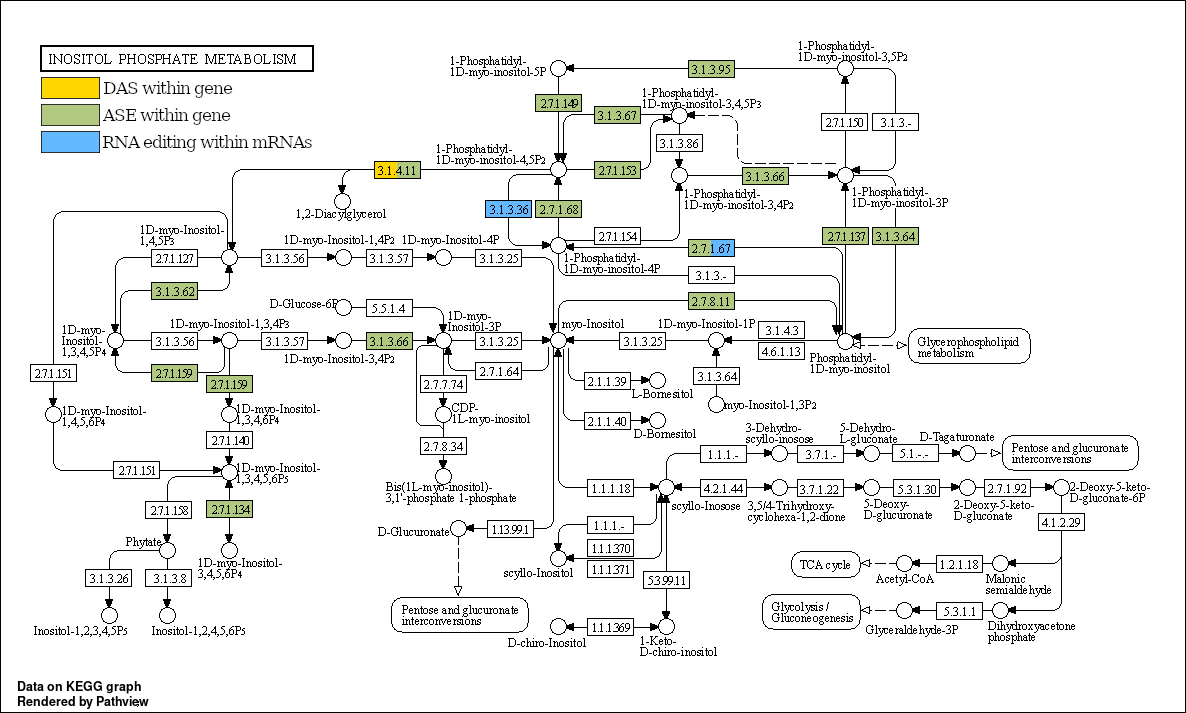

Supplement: Supplementary file 1 [file ijms-24-05946-s001.zip › Supplementary materials/Figure S12.png]

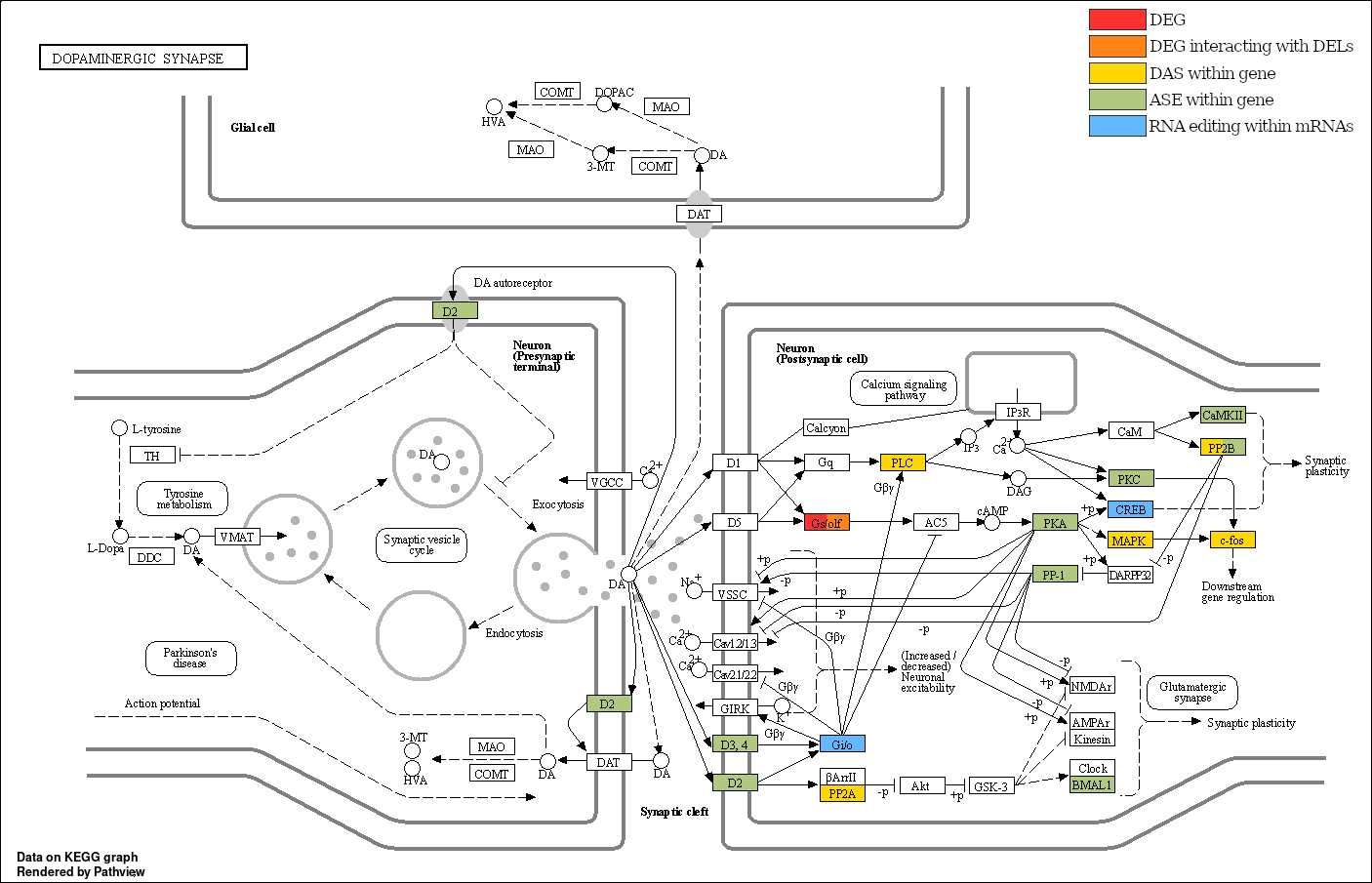

Supplement: Supplementary file 1 [file ijms-24-05946-s001.zip › Supplementary materials/Figure S13.png]

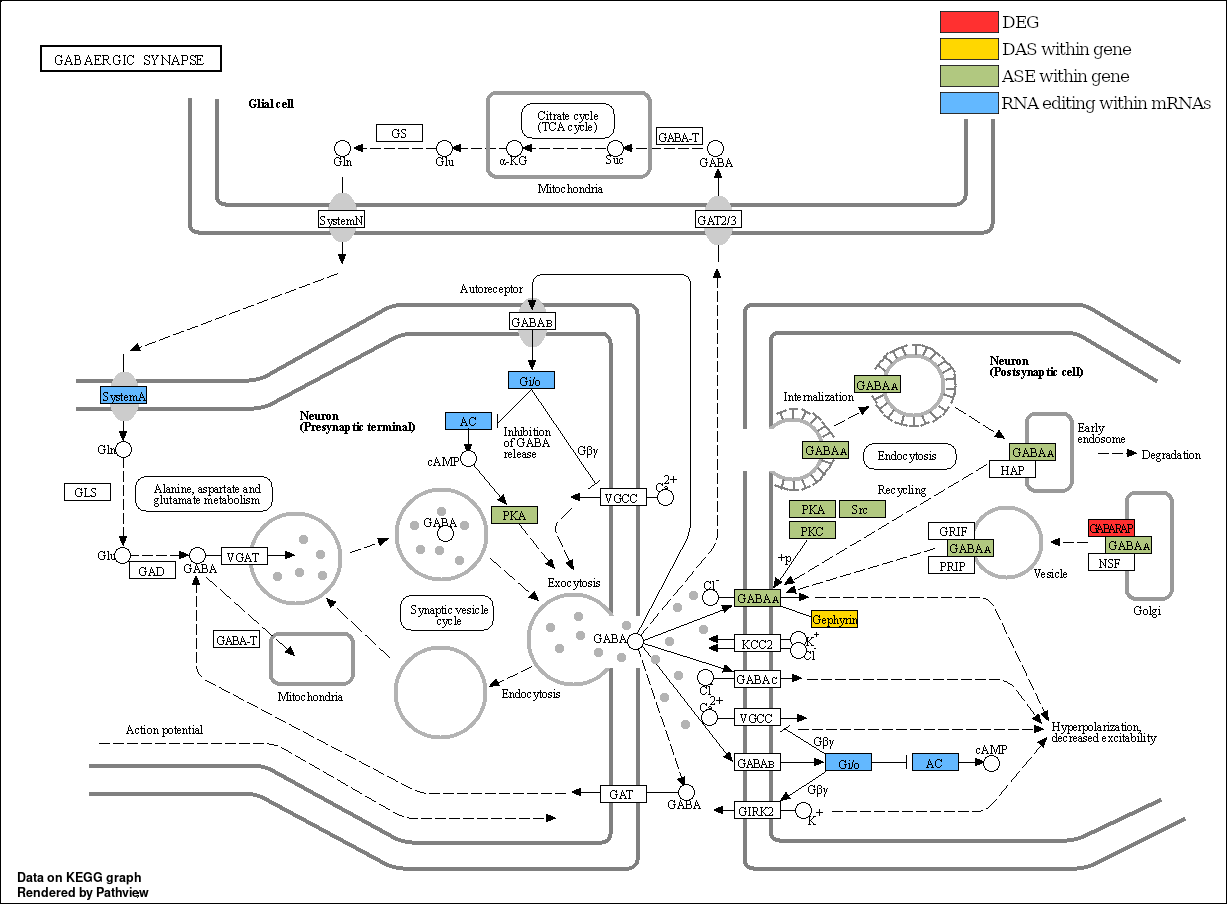

Supplement: Supplementary file 1 [file ijms-24-05946-s001.zip › Supplementary materials/Figure S14.png]

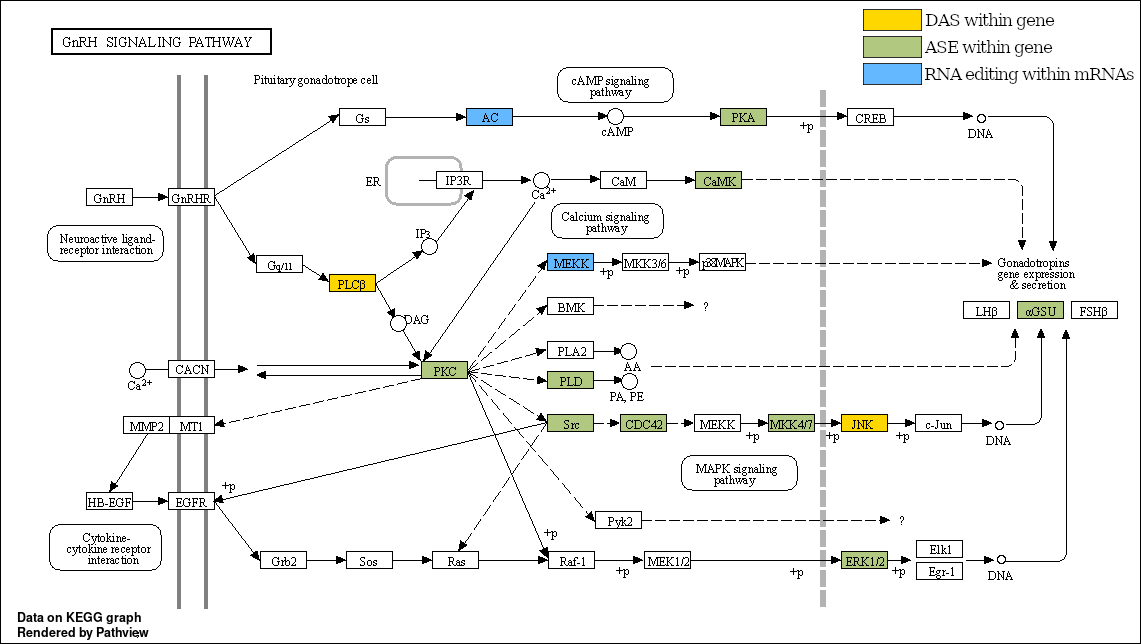

Supplement: Supplementary file 1 [file ijms-24-05946-s001.zip › Supplementary materials/Figure S15.png]

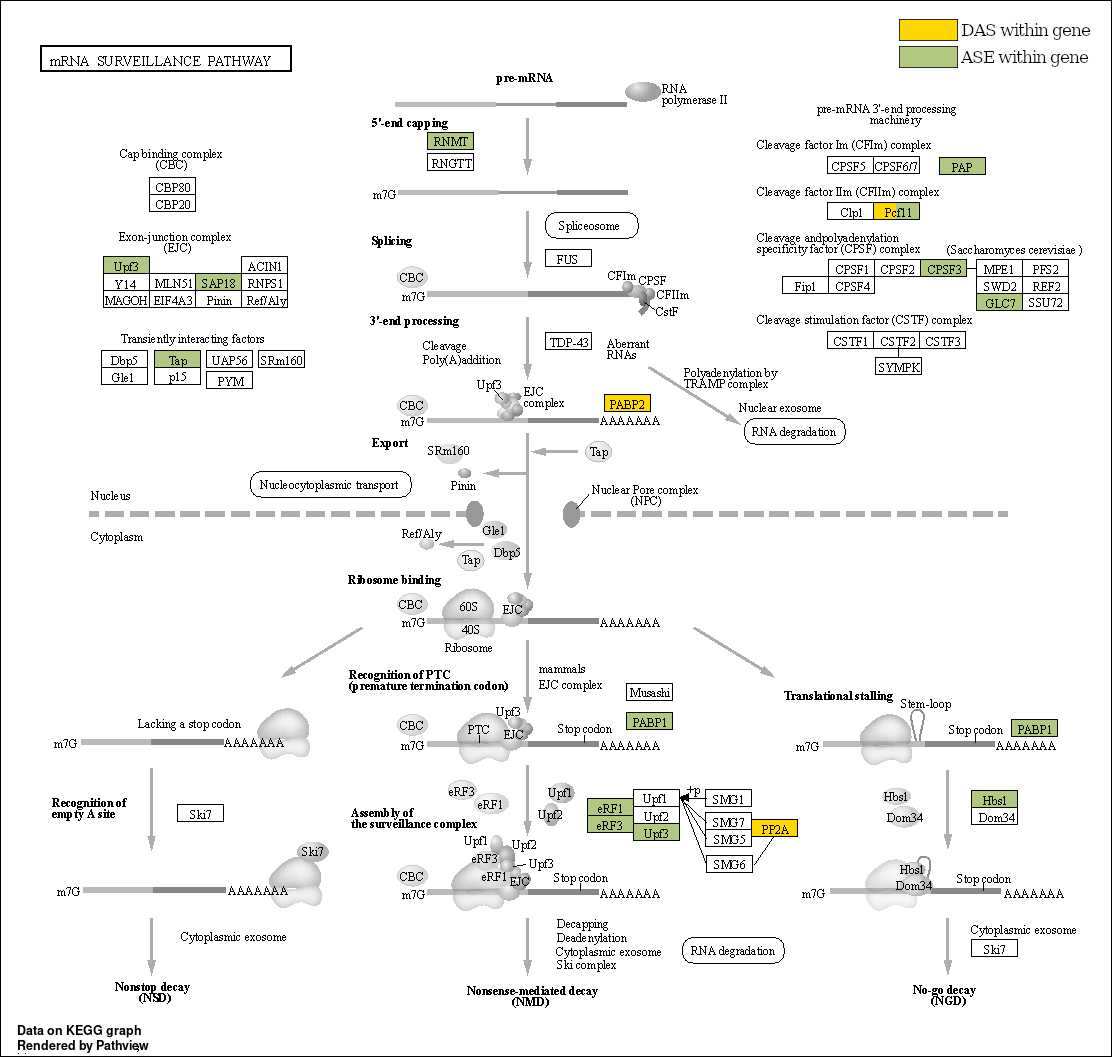

Supplement: Supplementary file 1 [file ijms-24-05946-s001.zip › Supplementary materials/Figure S16.png]

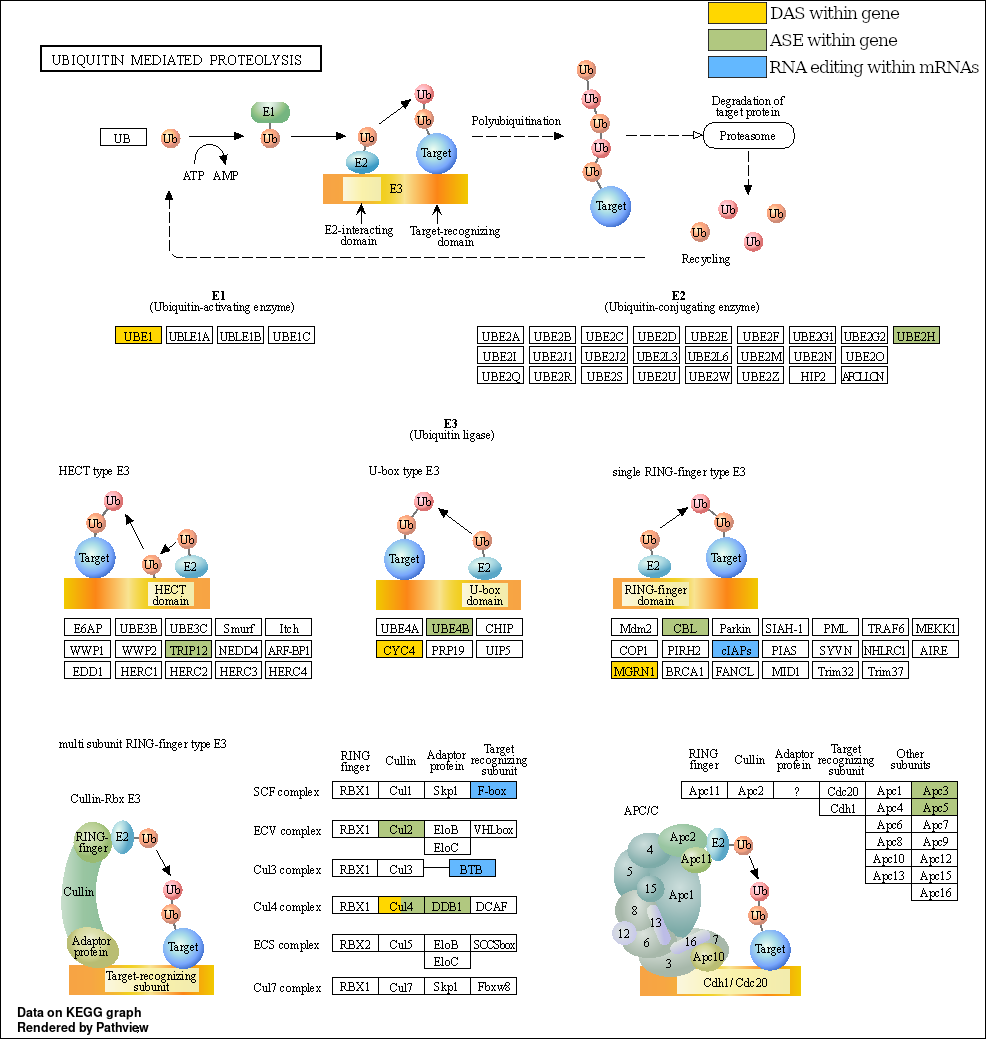

Supplement: Supplementary file 1 [file ijms-24-05946-s001.zip › Supplementary materials/Figure S17.png]

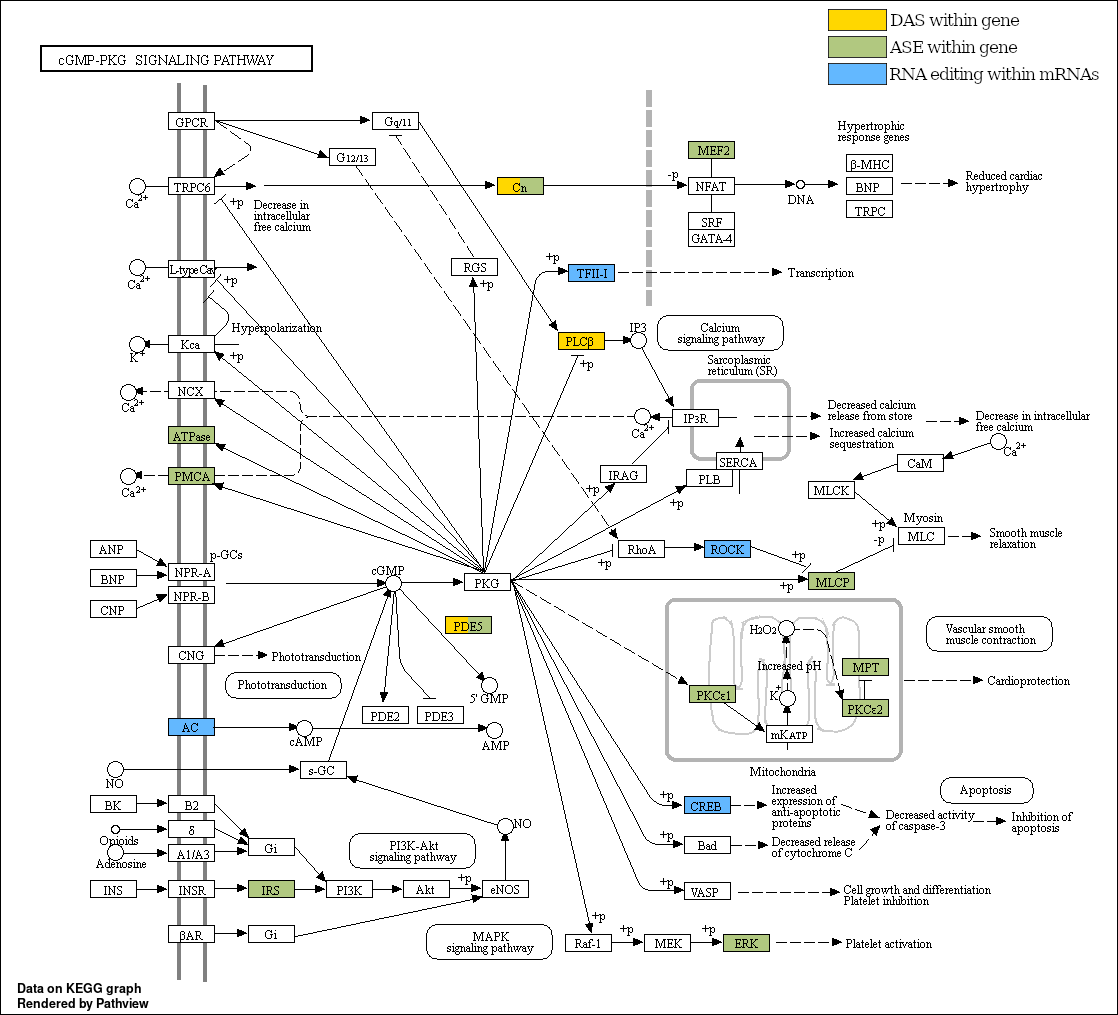

Supplement: Supplementary file 1 [file ijms-24-05946-s001.zip › Supplementary materials/Figure S18.png]

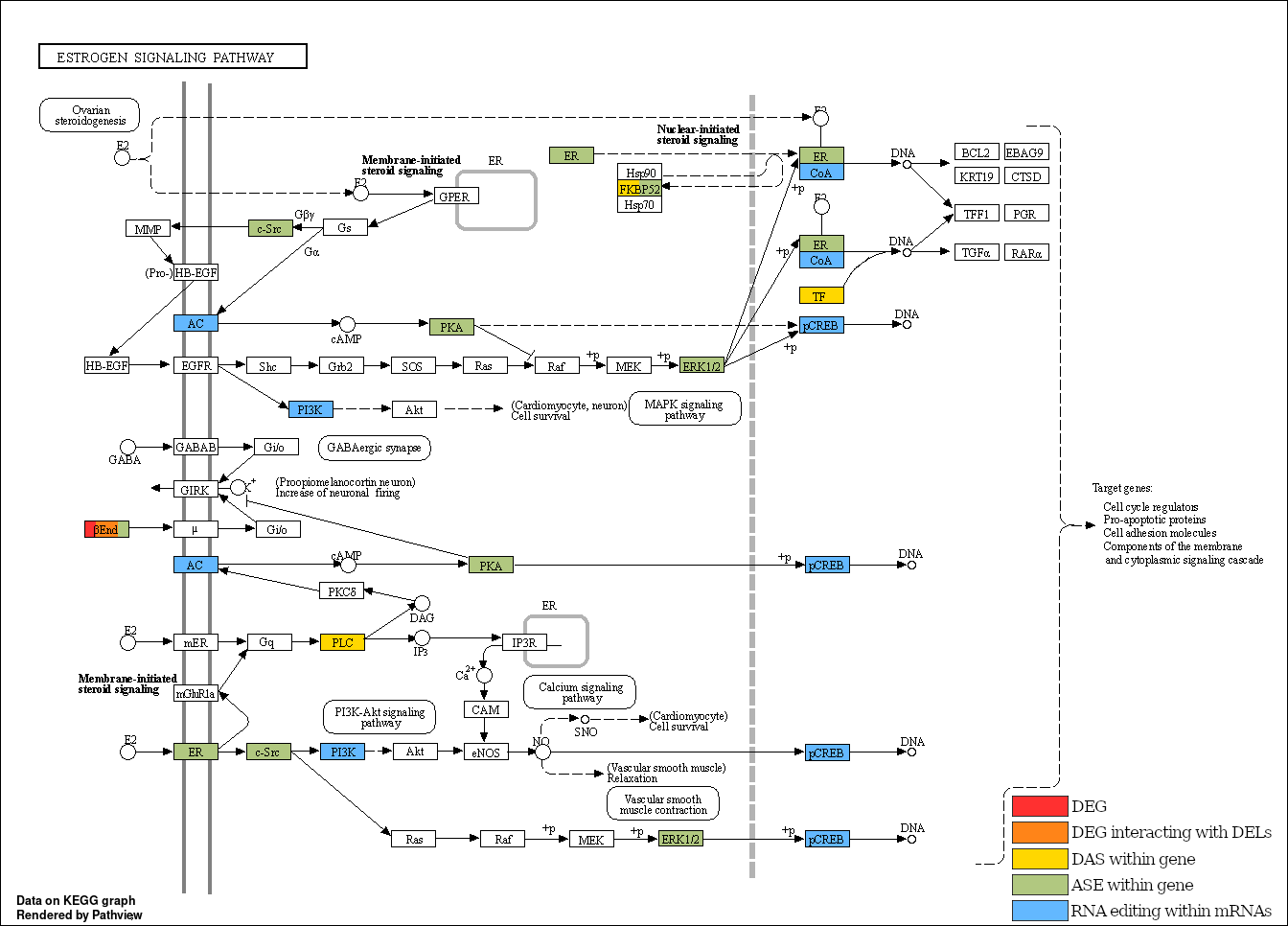

Supplement: Supplementary file 1 [file ijms-24-05946-s001.zip › Supplementary materials/Figure S19.png]

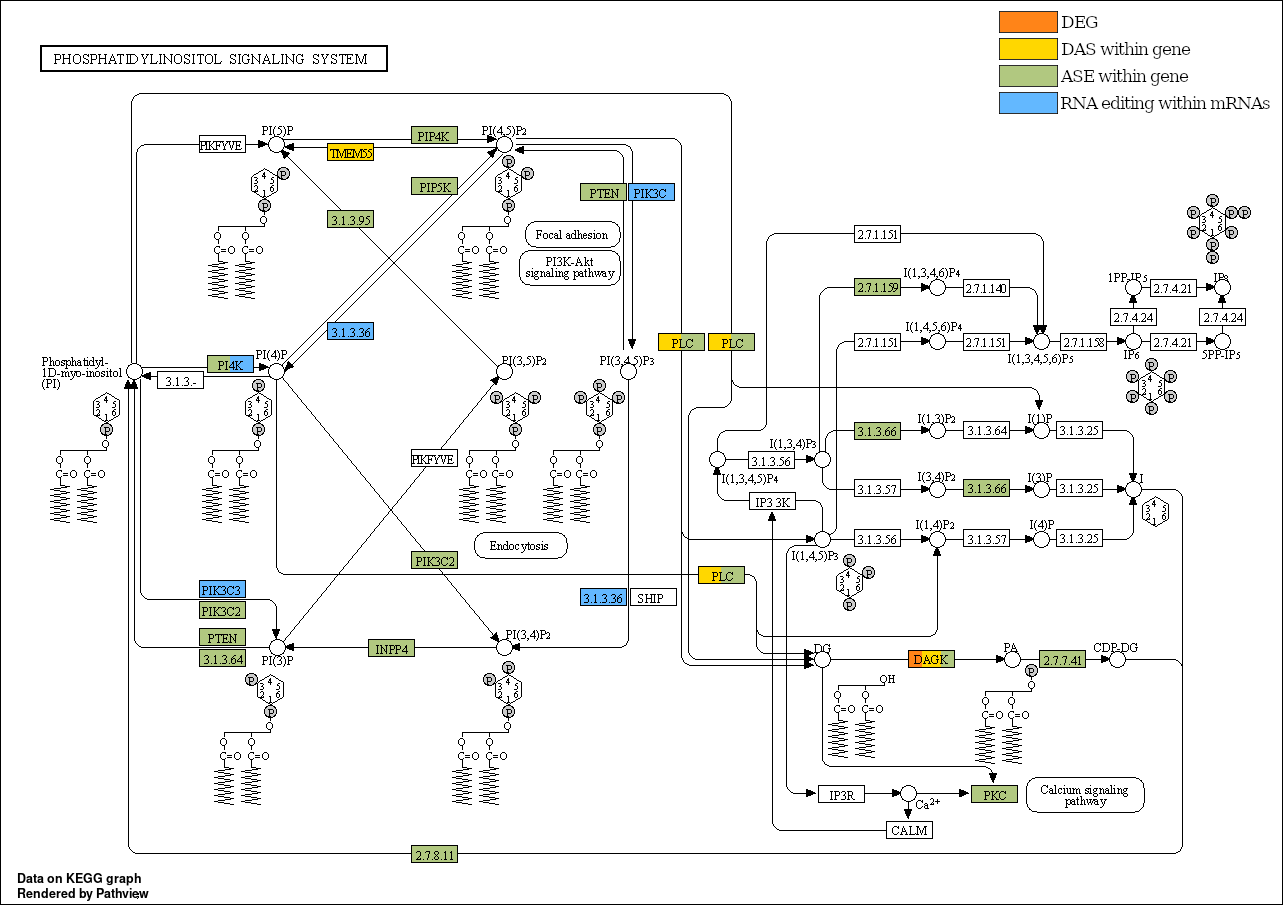

Supplement: Supplementary file 1 [file ijms-24-05946-s001.zip › Supplementary materials/Figure S2.png]

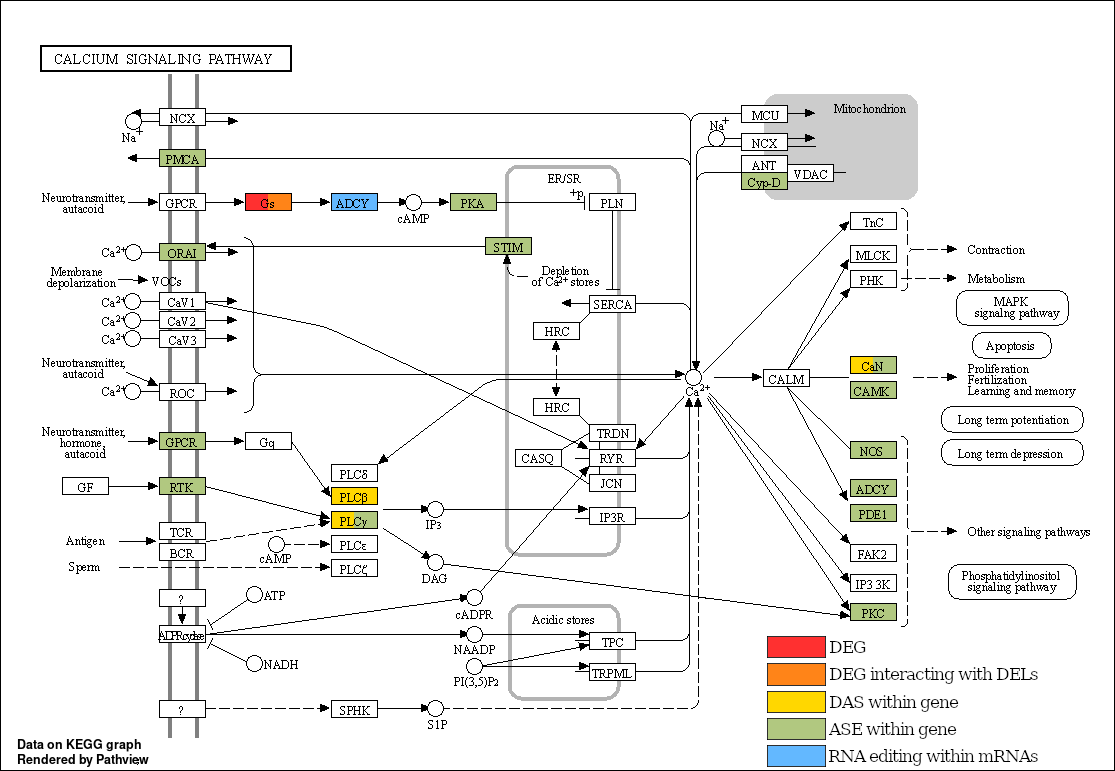

Supplement: Supplementary file 1 [file ijms-24-05946-s001.zip › Supplementary materials/Figure S20.png]

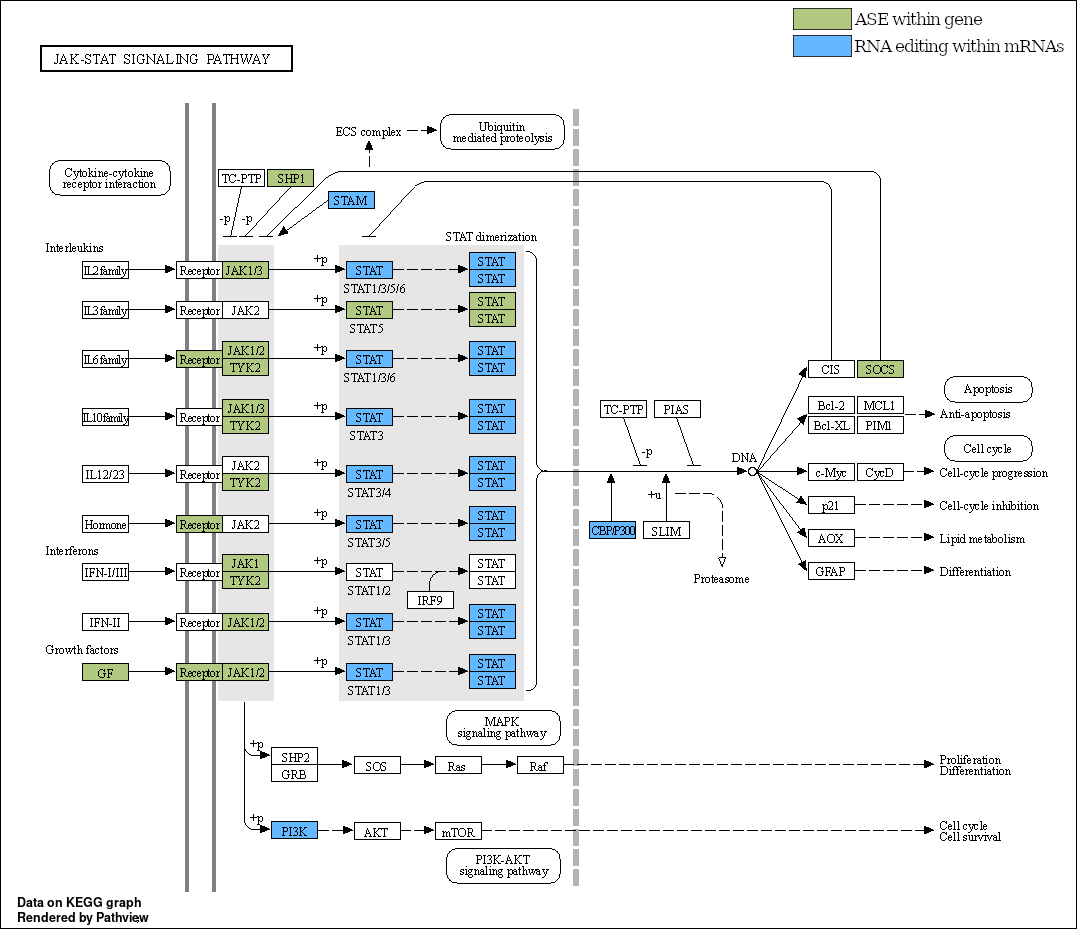

Supplement: Supplementary file 1 [file ijms-24-05946-s001.zip › Supplementary materials/Figure S21.png]

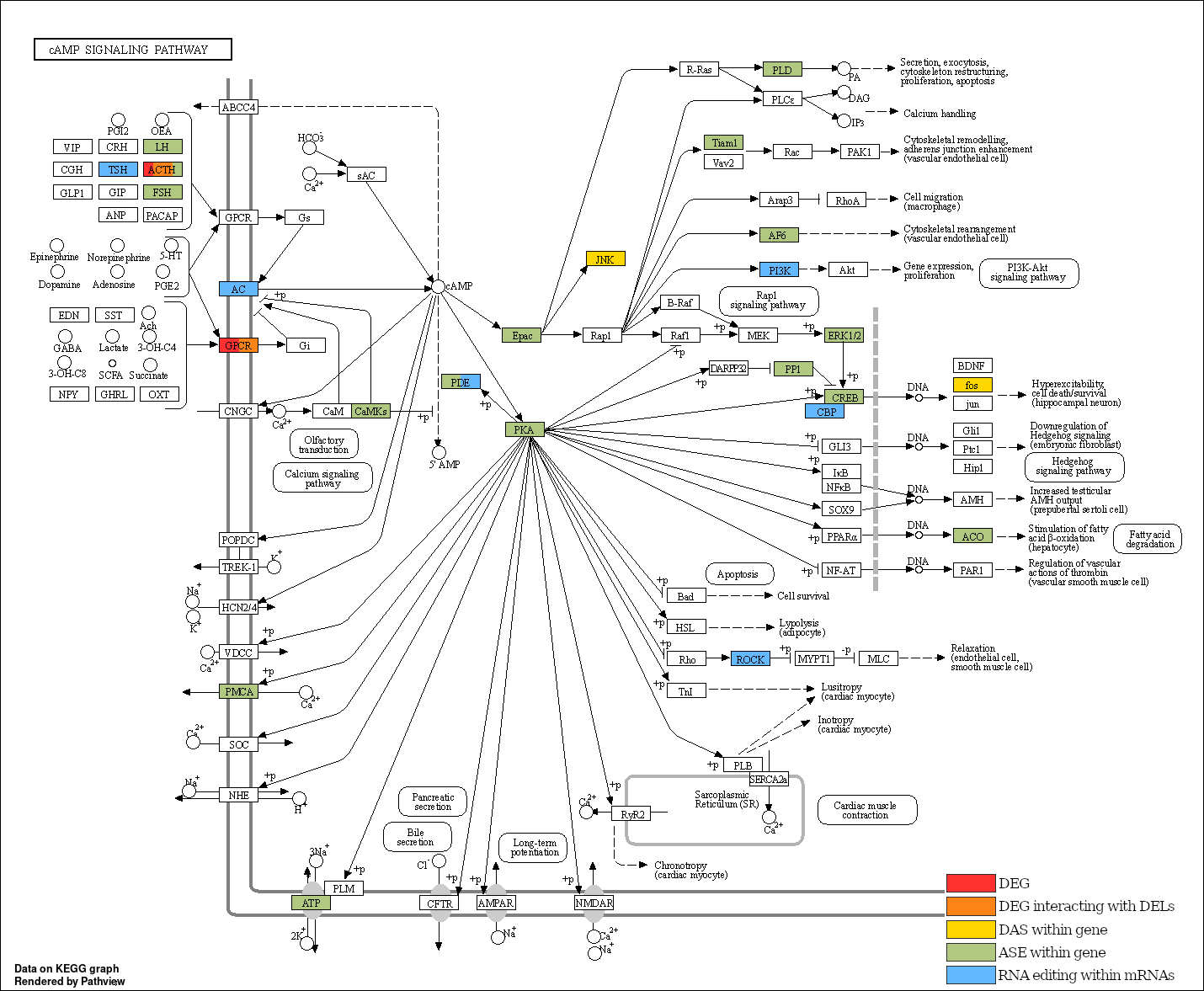

Supplement: Supplementary file 1 [file ijms-24-05946-s001.zip › Supplementary materials/Figure S3.png]

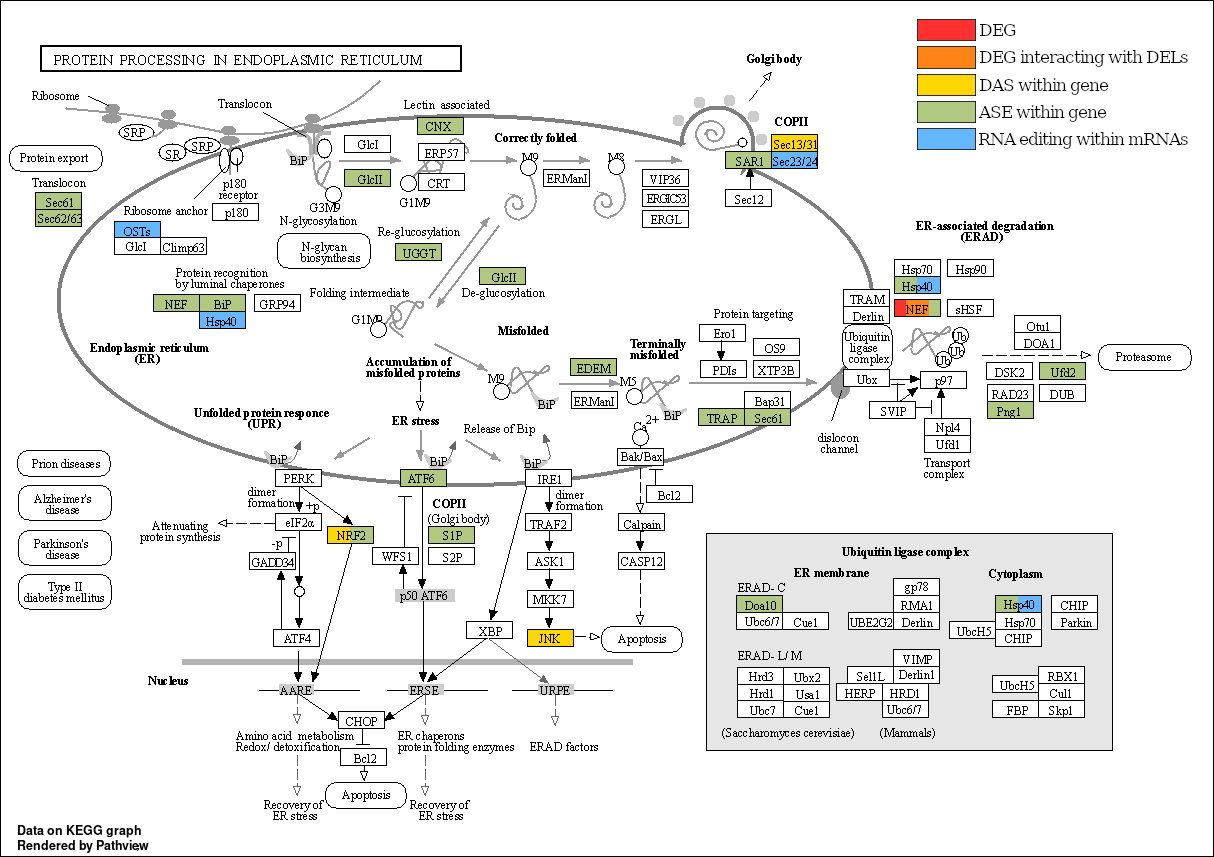

Supplement: Supplementary file 1 [file ijms-24-05946-s001.zip › Supplementary materials/Figure S4.png]

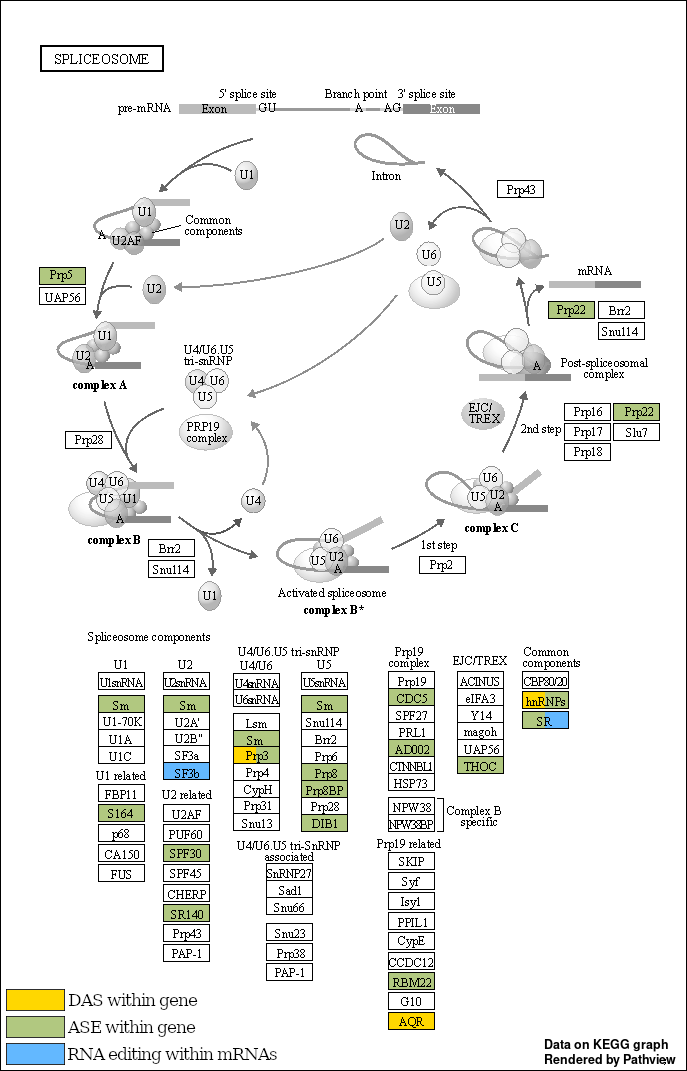

Supplement: Supplementary file 1 [file ijms-24-05946-s001.zip › Supplementary materials/Figure S5.png]

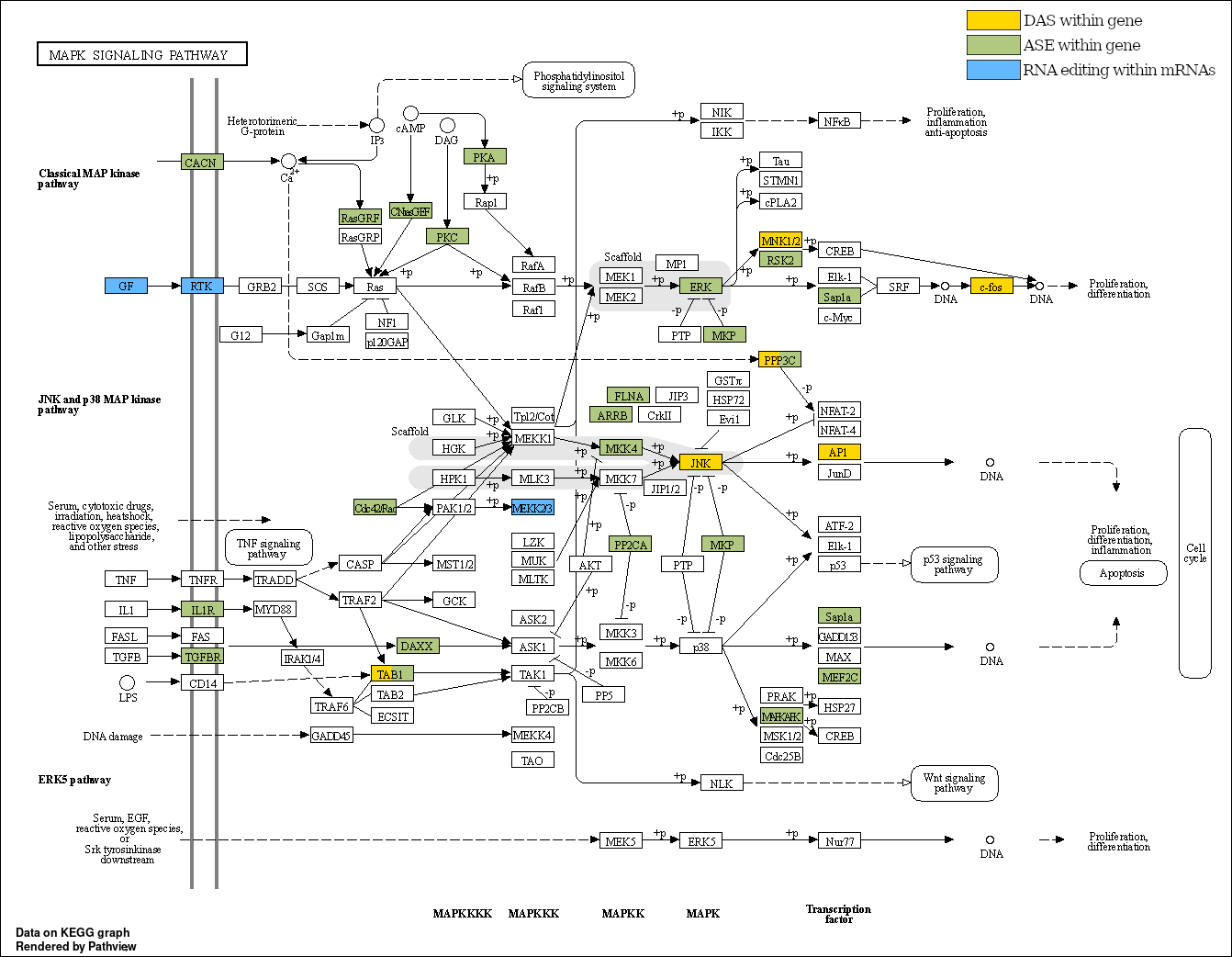

Supplement: Supplementary file 1 [file ijms-24-05946-s001.zip › Supplementary materials/Figure S6.png]

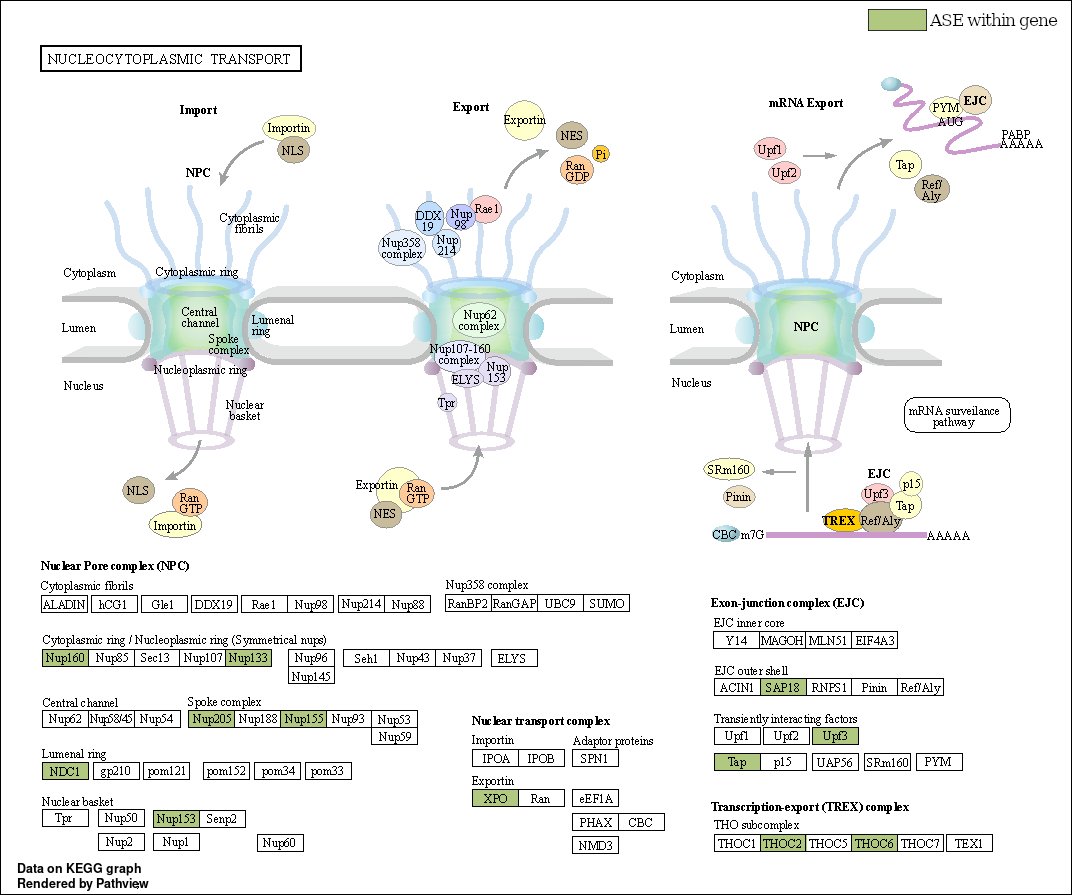

Supplement: Supplementary file 1 [file ijms-24-05946-s001.zip › Supplementary materials/Figure S7.png]

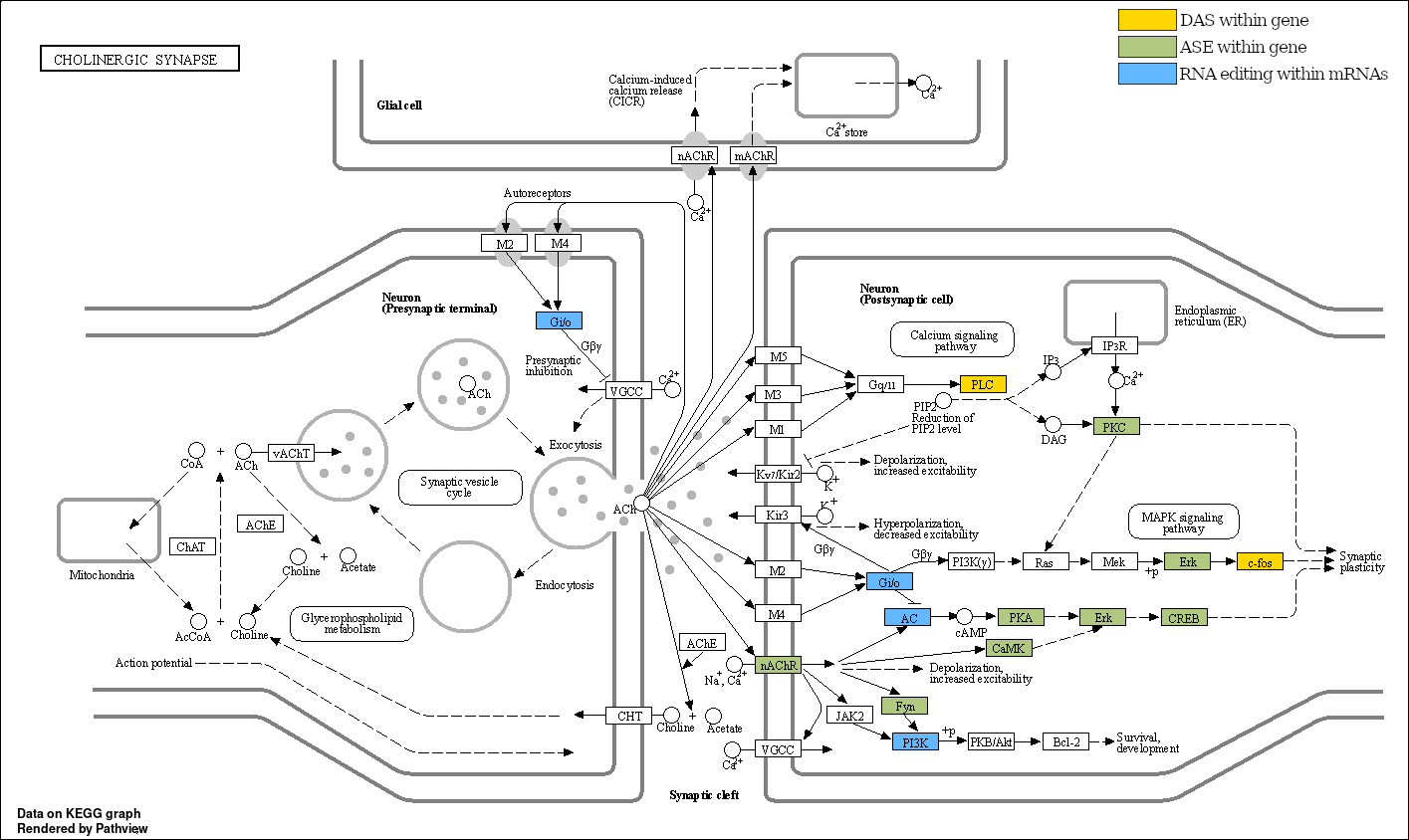

Supplement: Supplementary file 1 [file ijms-24-05946-s001.zip › Supplementary materials/Figure S8.png]

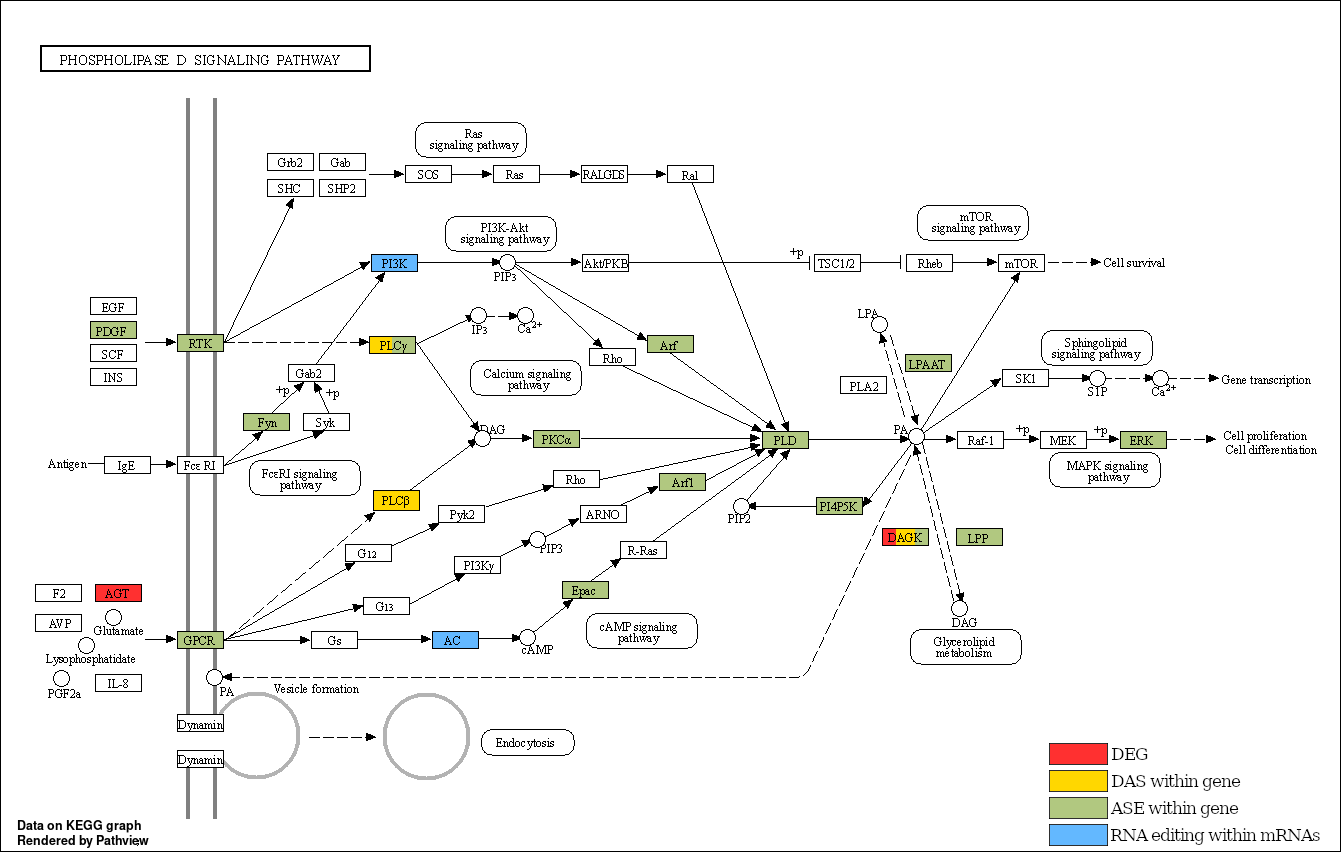

Supplement: Supplementary file 1 [file ijms-24-05946-s001.zip › Supplementary materials/Figure S9.png]
